# 2D versus 3D tumor-on-chip models to study the impact of tumor organization on metabolic patterns in vitro

**DOI:** 10.1038/s41598-025-03504-8

**Published:** 2025-06-04

**Authors:** Paula Guerrero-López, Ana Martín-Pardillos, Javier Bonet-Aleta, Andrea Mosseri, Jose L. Hueso, Jesus Santamaria, Jose Manuel Garcia-Aznar

**Affiliations:** 1 Multiscale in Mechanical and Biological Engineering (M2BE), Aragon Institute of Engineering Research (I3A), University of Zaragoza, Mariano Esquillor s/n, 50018 Zaragoza, Spain; 2 Instituto de Nanociencia y Materiales de Aragon (INMA), CSIC-Universidad de Zaragoza, Campus Rio Ebro, Edificio I+D, C/Mariano Esquillor, s/n, 50018 Zaragoza, Spain; 3 Department of Chemical Engineering and Environmental Technology (IQTMA), University of Zaragoza, 50018 Zaragoza, Spain; 4 Networking Research Center in Biomaterials, Bioengineering and Nanomedicine (CIBER-BBN), Instituto de Salud Carlos III, 28029 Madrid, Spain; 5 Instituto de Investigación Sanitaria (IIS) de Aragón, Avenida San Juan Bosco, 13, 50009 Zaragoza, Spain; 6 Yusuf Hamied Department of Chemistry, University of Cambridge, Cambridge, CB2 1TN UK; 7 Escuela Politécnica Superior, Universidad de Zaragoza, Crta. de Cuarte s/n, 22071 Huesca, Spain

**Keywords:** 2D cell culture, 3D cell culture, Metabolism, Spheroid, Glucose, Cancer, Cancer metabolism, Cancer models, Biomaterials - cells

## Abstract

Despite the limitations of in vitro models to investigate cancer cell metabolism, their study can provide new insights essential for understanding tumorigenesis and effectively aiding in the development of novel therapies. The innovative tumor-on-chip models offer a more physiologically relevant platform than the traditional 2D cultures. These 3D cultures incorporate cell–cell and cell–matrix interactions, as well as diffusion dynamics through both the matrix and tumor spheroid, modeling in vivo diffusion within the tumor. Therefore, this work focuses on a quantitative comparison between 2D and 3D cultures through a microfluidic chip that allows daily monitoring of cancer cell key metabolites such as glucose, glutamine and lactate, unveiling critical differences. Our analysis reveals reduced proliferation rates in 3D models, likely due to limited diffusion of nutrients and oxygen. Additionally, 3D cultures showed distinct metabolic profiles, including elevated glutamine consumption under glucose restriction and higher lactate production, indicating an enhanced Warburg effect. The microfluidic chip enabled continuous monitoring, revealing increased per-cell glucose consumption in 3D models, highlighting fewer but more metabolically active cells than in 2D cultures. These findings underscore the importance of using microfluidic-based 3D models to provide a more accurate representation of tumor metabolism and progression compared to traditional 2D cultures.

**Supplementary Information:**

The online version contains supplementary material available at 10.1038/s41598-025-03504-8.

## Introduction

The behavior of cancer cells in vitro is often studied to gain invaluable knowledge about cellular mechanisms of tumor proliferation, response to environmental conditions, and as a first step in the development of cancer therapies. However, these investigations are influenced by a multitude of factors, including extracellular matrix biophysical properties, cell–cell interactions, and nutrient availability^1,2^. Traditional two-dimensional (2D) cell cultures are widely used in cell-based assays since they are well-stablished and constitute an easy methodology to test multiple and different conditions. However, the 2D cell culture method also presents severe limitations and fails to accurately mimic the physiological conditions encountered by cancer cells within solid tumors^3^. This can lead to misleading data, especially in drug discovery where only about 10% of compounds progress successfully from 2D cell culture tests to clinical trials^4–6^. In contrast, three-dimensional (3D) cell culture models offer a more physiologically relevant environment by allowing cells to interact between them and with the extracellular matrix (ECM), leading to the formation of multicellular spheroids that better resemble the architecture of in vivo tumors^7–10^. Recent studies have demonstrated that cancer cells cultured in 3D exhibit altered metabolic profiles and growth kinetics compared to their 2D counterparts, suggesting that the dimensionality of the cellular environment plays a crucial role in modulating cancer cell behavior and regulating tumor cell organization^3,11^.

The architecture of cell culture models influences both metabolic profiles and cytotoxicity responses. In 2D cultures, cells receive nutrients uniformly, whereas in 3D cultures, nutrient diffusion is limited, creating distinct microenvironments. This affects cell evolution and fate, with 2D cultures favoring a predominantly proliferative population, while 3D cultures contain cells at various stages, including proliferation, quiescence, apoptosis, hypoxia, and necrosis^3^. The metabolic phenotype of different cancer cell lines has been studied comparing 2D cultures and 3D spheroids, revealing significant differences in glucose metabolism, related to glycolysis and oxidative phosphorylation. Spheroids show a higher ATP-linked respiration in normal nutrient conditions which shift to higher non-aerobic ATP production in the absence of glucose. Nevertheless, the metabolic phenotype shift from 2 to 3D culture is different in each cell line and tumor source^11^. Additionally, HCT116 spheroids show reduced sensitivity to ATP synthase inhibition, which has been linked to metabolic differences affecting chemotherapeutic responses in 2D and 3D cultures^12^.

At genetic level, gene expression has shown significant differences between 2 and 3D cultures in prostate cancer cell lines. Genes as ANXA1 (recently reported as a possible tumor suppressor gene), CD44 (cell-surface glycoprotein involved in cell–cell interactions, cell adhesion and migration), OCT4 and SOX2 genes (related with self-renewal) and ALDH1 were altered in 3D cultures^3^. Genes involved in drug metabolism such as CYP2D6, CYP2E1, NNMT, and SLC28A1 were slightly upregulated in the 3D hepatocellular carcinoma cell cultures, while certain genes such as ALDH1B1, ALDH1A2, and SULT1E1 were downregulated^13^. These results shown how gene expression profile is strongly influenced by the culture dimensionality.

There is a wide variety of studies about cancer metabolism using 3D cancer models^14^ through different techniques that include magnetic bioprinting^3^, cell aggregation by Cellbed^15^, U-Bottom plates, Hanging Drop method^11^ or tissue culture flasks with low attachment coating^16^. All these methods are based on spheroid formation through artificial cell aggregation, where an external force such as magnetic, centrifugation or gravity makes cancer cell clump together into a spheroid. However, our approach is based on seeding individual cells inside a collagen-based hydrogel that mimics ECM^17^ in order to induce cells proliferation and understand how they self-organize along the hydrogel microarchitecture. These cancer models are developed in microfluidic-based chips. Microfluidics applied to cell culture has great advantages such as flexibility in the design of the culture device, a low number of cells needed or the possibility of real-time analysis within the devices^18^. This method of cell culture allows the creation of microbioreactors in which the cells are embedded in this hydrogel, where mechanical forces help them to clump together and grow into spheroids, thus providing a model that imitates the process of tumorigenesis that occurs at the physiological level^19–21^.

It is well known that glucose metabolism is altered in tumor cells, resulting in the well-known Warburg effect^22,23^. Furthermore, the tumor spheroid, even if it is composed of one single type of cells, is not homogeneous. On the contrary, heterogeneity can be considerable, with cells presenting different consumption patterns according to their location within the tumor, so it is also important to study these consumption dynamics^1,21,24^. Finally, glutamine is a key amino acid in tumor development, as it is the most abundant amino acid in blood and tissues, and alterations in its metabolism are very characteristic in cancer patients ^25^. Since depletion of glutamine in the host has adverse effects, it is important to study the regulation of glutamine metabolism in cancer as an alternative pathway to glucose metabolism.

**Fig. 1:**
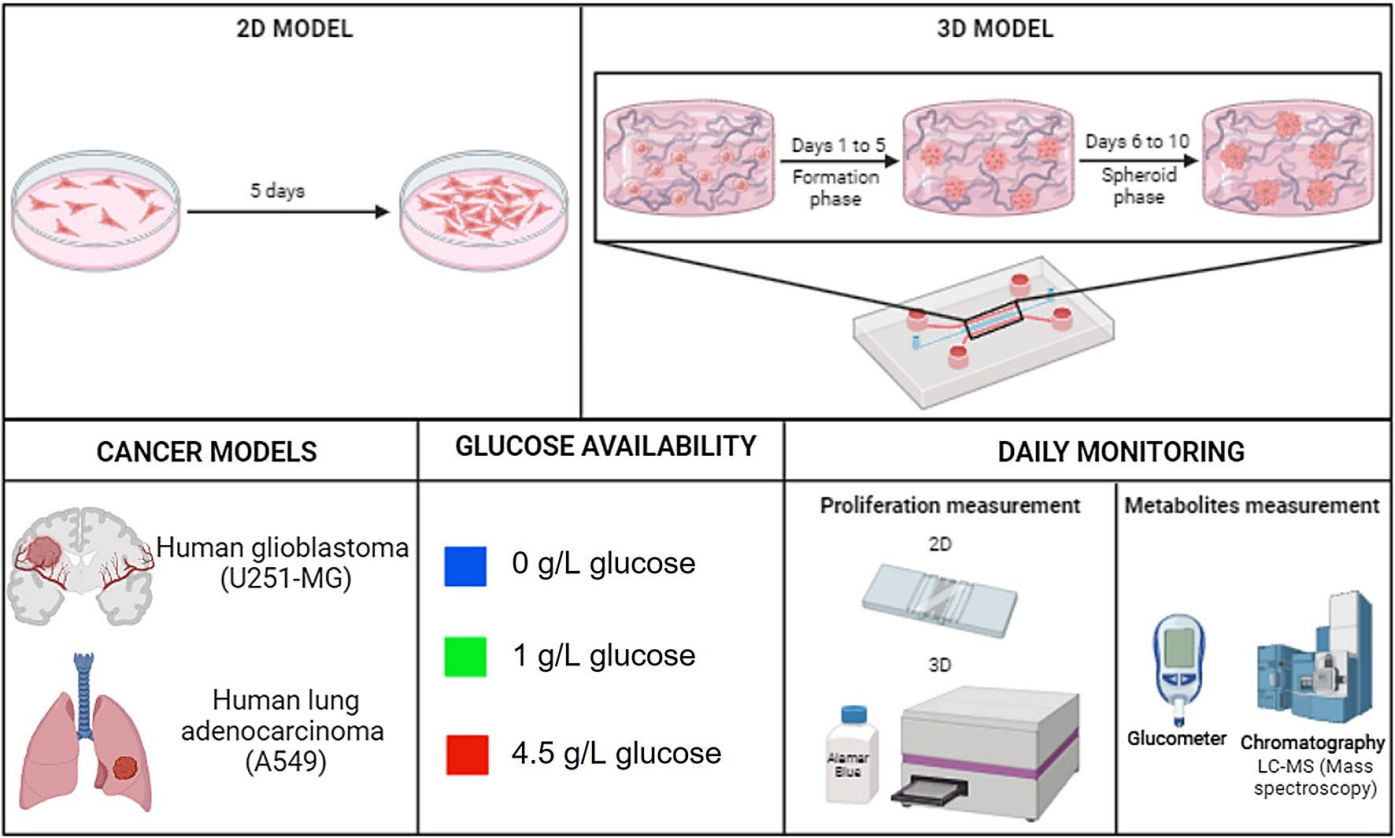
Scheme of experimental design. 2D and 3D models used (up), cell models tested (down-left), glucose conditions applied (down-center) and parameters measured (down-right). Created in BioRender. Guerrero-López, P. (2025) https://BioRender.com/sznsoy1.

In this work, by comparing 2D and 3D models, we seek to study differences on cancer cell metabolism, proliferation, organization and progression. A main focus is on glucose metabolism as glucose is a key nutrient of cancer cells^26^ and we study the differences observed in traditional 2D cell culture vs 3D architecture. Comparative time-evolution assays were performed in which proliferation, metabolically active cells, consumption of glucose and glutamine and production of lactate were measured in both U251-MG human glioblastoma and human lung adenocarcinoma A549 cell lines, as examples of cancer cells with diverse glucose requirements (Fig. 1). The evolution under different glucose concentration levels was compared to establish effects over cancer progression, tumor development and activation of alternative metabolic pathways.

## Results

### Cell proliferation is more glucose-dependent in 2D culture than in 3D

The pivotal role of glucose as the primary nutrient for cellular growth has long been established^26^, so our aim was to understand how variations in glucose availability can modulate cellular behavior in 2D and 3D conditions. To this end, we studied the proliferation patterns of two cellular lines with different glucose requirements under three distinct glucose conditions.

**Fig. 2:**
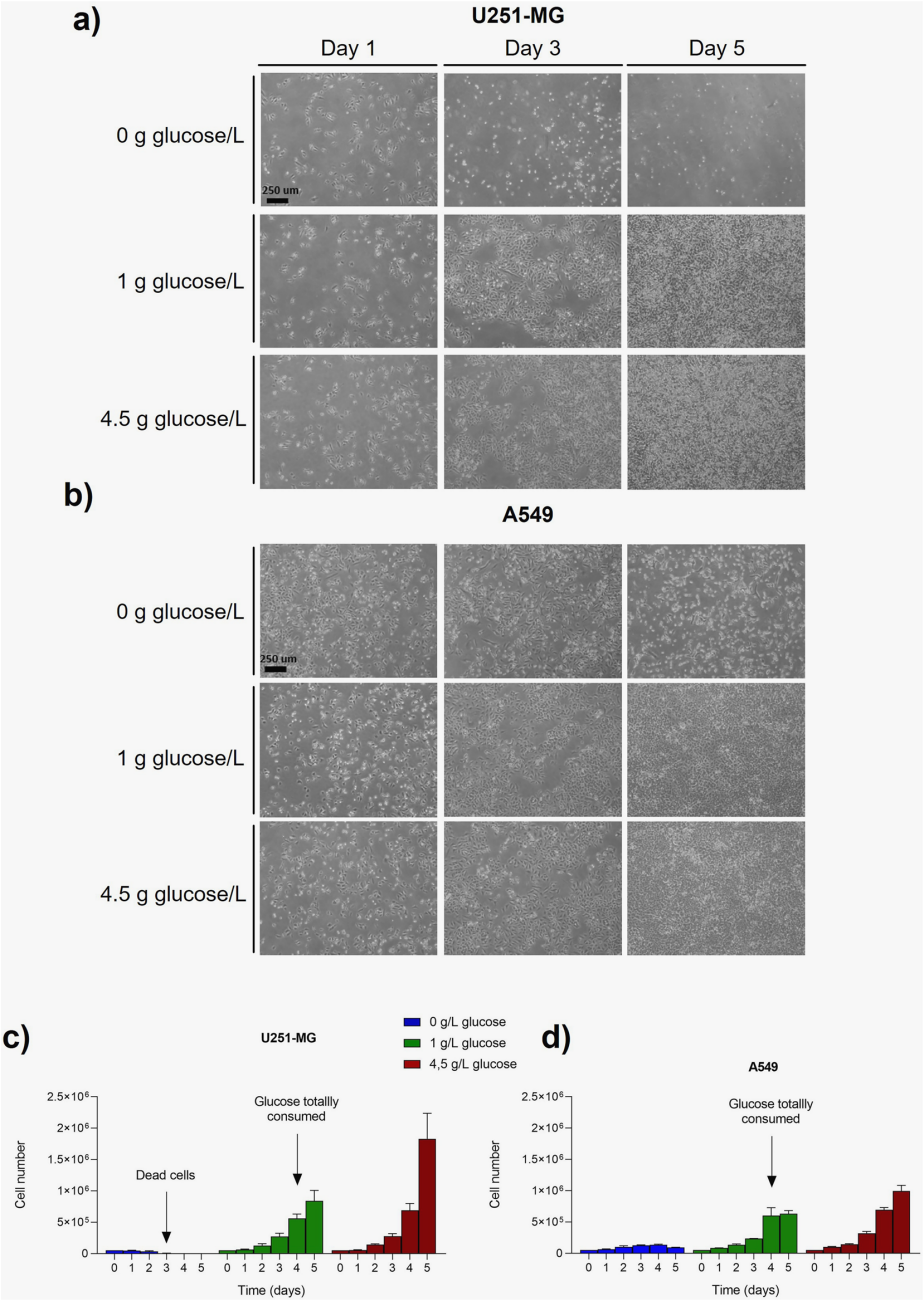
Proliferation characterization in 2D cell culture. Brightfield images of U251-MG (**a**) and A549 (**b**) development for 5 days were taken depending on glucose availability. Quantitative data of cell proliferation obtained from Neubauer counting for U251-MG (**c**) and A549 (**d**) cells lines.

To characterize the proliferation patterns in 2D (Fig. 2 and Tables S1 and S2), A549 and U251-MG cells were maintained for 5 days in culture, taking daily measurements of proliferation through image acquisition and Neubauer chamber counting. Similar growth was observed in brightfield microscopy for both glioblastoma (Fig. 2a) and lung adenocarcinoma (Fig. 2b) cells lines during 4 days when glucose was available, regardless of whether the glucose concentration was high or low. By day 5, both cell lines reached confluence, although glioblastoma cells displayed a notably higher proliferation rate. However, when glucose was removed from culture medium, cells stopped their proliferation, even displaying a decrease in cell number, due to cell death. Looking at quantitative data, glioblastoma cells behavior was translated into an exponential growth when glucose was available, although it decelerated when this nutrient was totally consumed in the condition of low glucose (Fig. 2c). When cells were not supplied with this nutrient, U251-MG were able to attach to the plate and maintain cell viability for 24 h (up to day 1), but they were not able to divide. At day 2 they started reducing the cell number due to cell death and there were not viable cells at day 3 of culture. In contrast, A549 were able to survive till day 5, but with a decrease in proliferation (Fig. 2d), however cell number started to reduce at day 4. When cultured with glucose, proliferation rates are similar to those of U251-MG ones up to day 4.

**Fig. 3:**
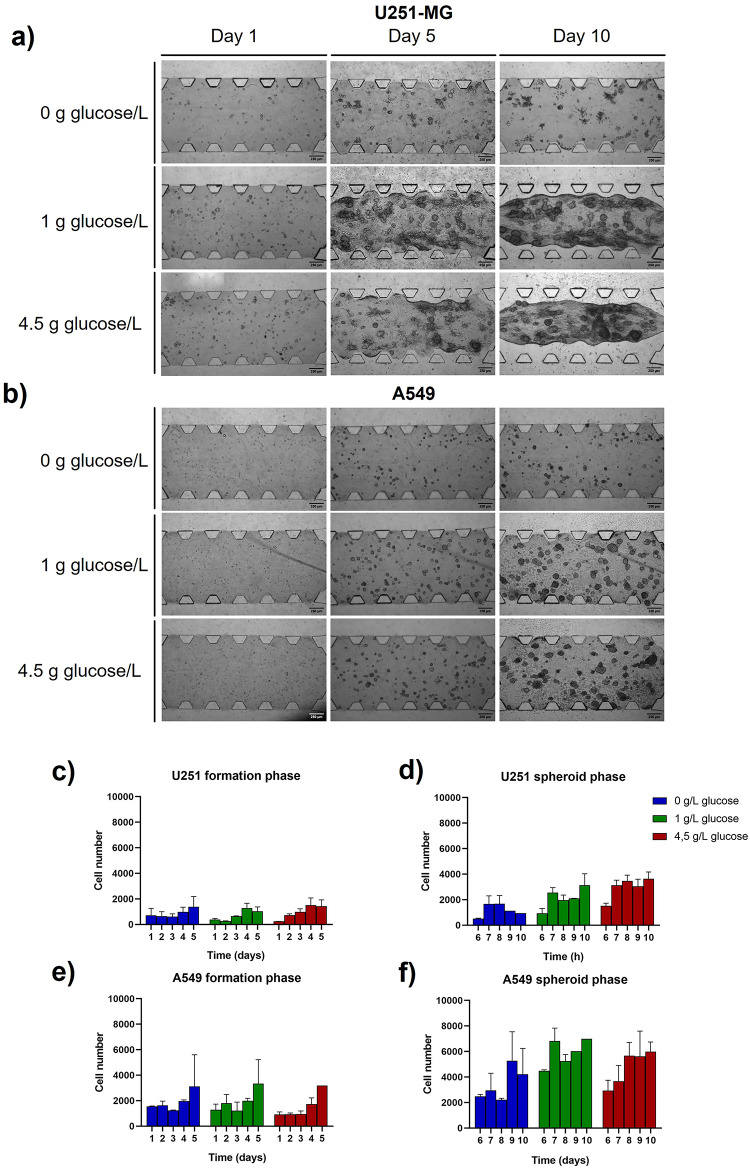
Proliferation characterization in 3D cell culture. Brightfield images of U251-MG (**a**) and A549 (**b**) development for 10 days were taken depending on glucose availability inside the microfluidic device. Quantitative data of cell proliferation: U251-MG in both formation (**c**) and spheroid (**d**) phase, and A549 in both formation (**e**) and spheroid (**f**) phase.

For 3D cultures assays, same cells and glucose conditions were used meanwhile culture time was extended up to 10 days (Fig. 3 and Tables S3 and S4). This allowed us to study 3D cancer cell metabolism in two phases as the first 5 days correspond to spheroid formation and, from day 6 to 10, the culture mimics tumor maintenance and growth. Similarly to 2D culture, cell culture pictures revealed no differences in proliferation between high or low glucose concentration for both, U251-MG (Fig. 3a) and A549 (Fig. 3b) cells. However, although a strong decrease in proliferation could be observed under glucose deprivation at endpoint (spheroid phase), no significant differences were detected by the end of the formation phase (day 5), particularly in A549 cells. This suggests that the activation of alternative metabolic pathways allows cells to survive and proliferate under glucose deprivation, although it prevents the development of large tumor structures as observed in glucose-rich conditions. These results revealed that cells in 3D morphology under glucose deprivation were able to survive and proliferate longer than with 2D methods. It is also noteworthy that the hydrogel deformation often observed during U251-MG growth in 3D conditions due to the mechanical stress developed, disappeared when cells were not supplied with glucose. In order to ensure no affection over the results by this ECM detachment, a validation experiment using a different surface coating (polydopamine) in the microfluidic chip was performed and no significant differences were revealed (Figure S1). For quantitative data, the number of cells metabolically active was obtained with Alamar Blue Reagent. In this way, alterations in culture development during the formation phase were not remarkable in glioblastoma cells (Fig. 3c), where the patterns observed were similar for high and low glucose levels and only slight differences were observed in case of no glucose culture. However, in the spheroid phase (Fig. 3d) significant differences in metabolically active cells patterns through time were shown as a function of glucose availability. Interestingly, lung adenocarcinoma cell counts had higher values than glioblastoma ones in both, formation (Fig. 3e) and spheroid (Fig. 3f) phase. In addition, proliferation patterns were similar in both phases displaying cyclic peaks of metabolic activity, but they were several folds higher in the spheroid phase.

### The available glucose in 2D cell culture strongly regulates the cell use of main metabolites

**Fig. 4:**
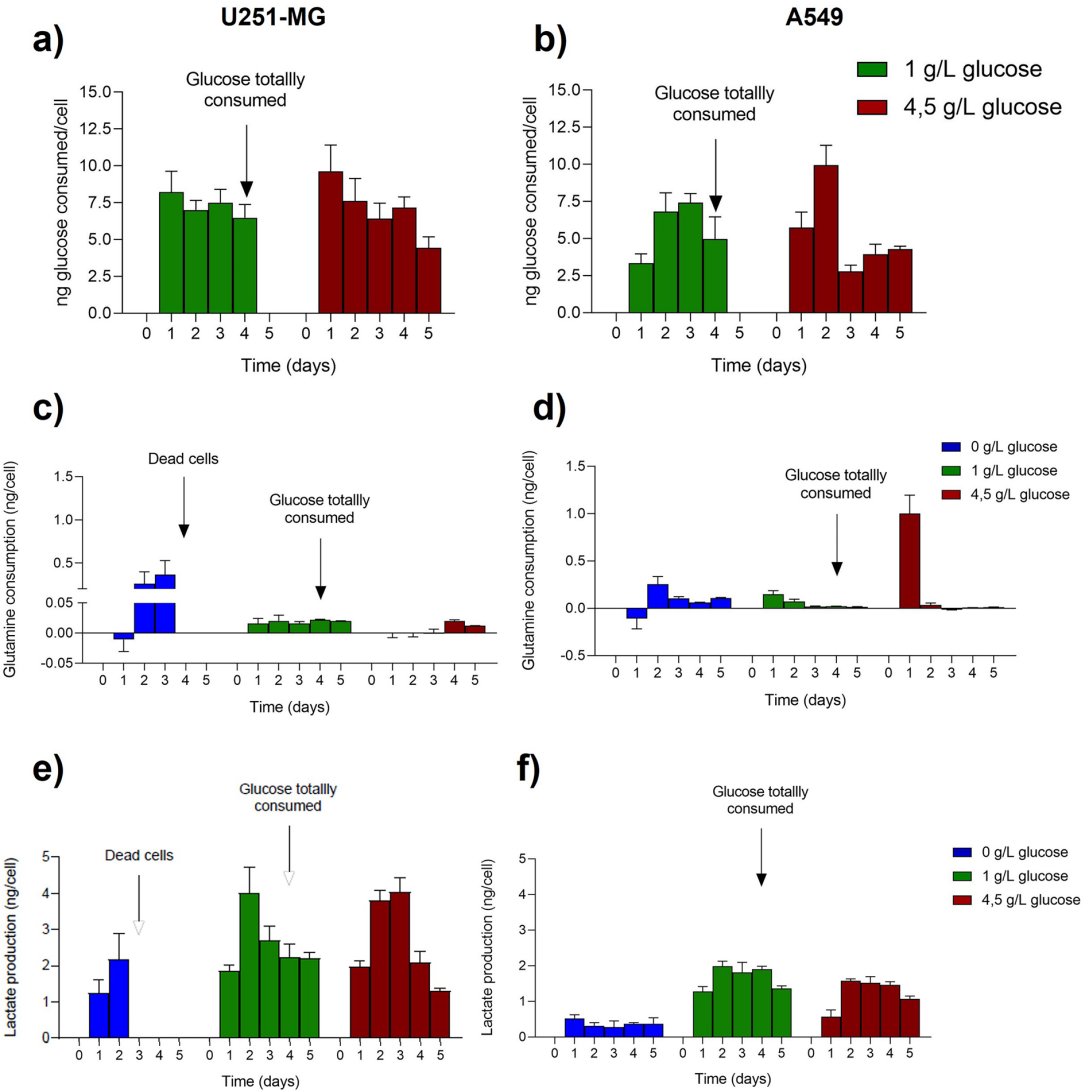
Metabolites evolution in time with cells in 2D cell culture for U251-MG (left) and A549 (right) cells lines. Glucose consumption normalized per cell for U251-MG (**a**) and A549 (**b**). Glutamine consumption normalized per cell for U251-MG (**c**) and A549 (**d**). Lactate production normalized per cell for U251-MG (**e**) and A549 (**f**).

In order to study metabolism in 2D cell culture, medium was collected daily and glucose, lactate and glutamine were quantified given their relation to glucose metabolism. In particular, lactate production was monitored given its role as the primary metabolite generated during anaerobic glycolysis, and glutamine as a key molecule often identified as an alternative to glucose to fuel the Krebs cycle in cancer cells^27^. In this way, the evolution of glucose, glutamine and lactate was obtained (Figure S2) and these data allowed us to establish a metabolite consumption/production per cell. Attending to glucose consumption, U251-MG cells displayed a downward trend indicating a decrease of glucose consumption as its level in the cell culture decreased (Fig. 4a and Table S5). This is in contrast with the behavior of A549 cells, where a clear trend cannot be discerned, and some consumption peaks appeared at different times (Fig. 4b and Table S6). At 24h, the low glucose consumption observed is likely due to the adaptation of these cells in the culture. An interesting fact is that for both cell lines similar glucose consumption patterns were obtained in the two (high and medium) glucose concentration scenarios, which is likely attributable to saturation of glucose transporters. When glucose availability exceeds the transport capacity of the cell through the membrane, the transporters operate at maximum rate, leading to a plateau in uptake regardless of further increases in external glucose concentration^28^.

Glutamine consumption per cell was notably higher in glioblastoma cells (Fig. 4c and Table S7) than in lung adenocarcinoma ones (Fig. 4d and Table S8). Interestingly, the use of this metabolite increased significantly for cells that had been cultured without glucose, especially in U251-MG cells. By contrast, lactate production was higher while there was a high amount of glucose in the microenvironment, indicating that cells were metabolizing glucose via the anaerobic pathway. As expected, U251-MG cells produced lactate (Fig. 4e and Table S9) at higher levels than A549 cells (Fig. 4f and Table S10) due to their high metabolic requirements. Again, the first day of experiment displayed a low production for both cell lines due to the already mentioned adaptation period, then increased by the second day, leading finally to a new reduction by the end of the experiment as glucose levels were depleted.

### Cellular metabolic requirements in 3D culture depend on the stage of tumor development

**Fig. 5:**
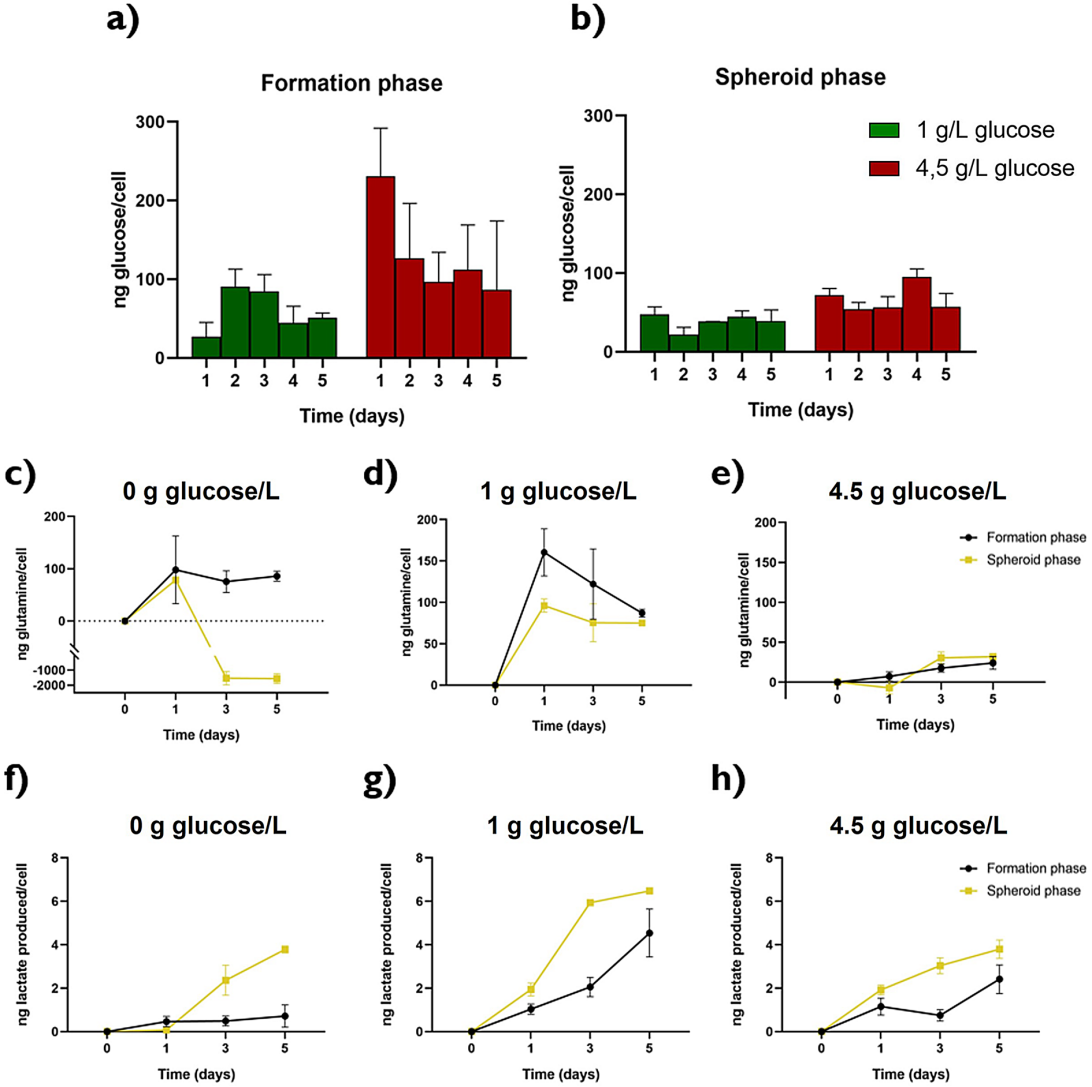
Metabolites evolution along time with cells in 3D cell culture for the U251-MG cell line. Glucose consumption per cell in formation (**a**) and spheroid (**b**) phase. Glutamine consumption per cell in both formation and spheroid phase for 0 g/L (**c**), 1 g/L (**d**) and 4.5 g/L (**e**) glucose. Lactate production per cell in both formation and spheroid phase for 0 g/L (**f**), 1 g/L (**g**) and 4.5 g/L (**h**) glucose.

A similar methodology was followed to study metabolism in our microfluidic devices with 3D models, although in this case the two phases of tumor development were taken into account. As explained above, preliminary experiments showed that the collagen matrix was able to reversibly adsorb glucose, decreasing the available concentration for cells. To take this into account glucose adsorption data in cell-free collagen were fitted to a Freundlich Isotherm Model that allowed us to consider the glucose trapping effect of collagen in in our study. By subtracting this glucose trapped, we were able to determine the actual glucose available for cells (Figure S9). The global glucose concentration presented a monotonic decrease for both cell lines revealing the progressive glucose consumption (Figure S3). However, the values of glucose consumption per cell in U251-MG cells indicate that it was higher in formation phase (Fig. 5a) than in spheroid one (Fig. 5b). Also, there was a dependency in glucose availability in the formation phase that was damped in the spheroid phase, where glucose consumption per cell was more similar in both concentration levels. See statistical analysis in Table S11.

The glutamine consumption patterns followed a similar trend to that seen in 2D cell culture, although the differences seem to be enhanced in 3D conditions. Thus, at the highest glucose concentration U251-MG cells had a really low glutamine consumption (Fig. 5e), but when glucose was reduced, glutamine consumption increased. Hence, in cells supplied with 1 g/L of glucose, a higher glutamine consumption was observed (Fig. 5d), and in the absence of glucose, glioblastoma cells also exhibited increased consumption of glutamine. However, this trend shifted during the spheroid phase, where negative values were obtained indicating glutamine synthesis in order to obtain an alternative fuel for metabolic routes essentials for cell surveillance under glucose restriction^25,29,30^ (Fig. 5c). Interestingly, U251-MG had major requirements of this metabolite in formation phase in all the conditions studied (Table S12). In contrast, when looking at lactate production it was always higher when the spheroids were already formed (Fig. 5f–h). Once again, the glucose intermediate concentration showed the higher values for this parameter, with the lowest ones at the culture with no glucose (Table S13). Under this condition, the lactate production peak at the end of the assay coincided with the stage where glutamine production was observed. Glutamine consumption and lactate production were clearly reflected in global evolution of metabolites through time in the cell culture (Figure S3).

**Fig. 6:**
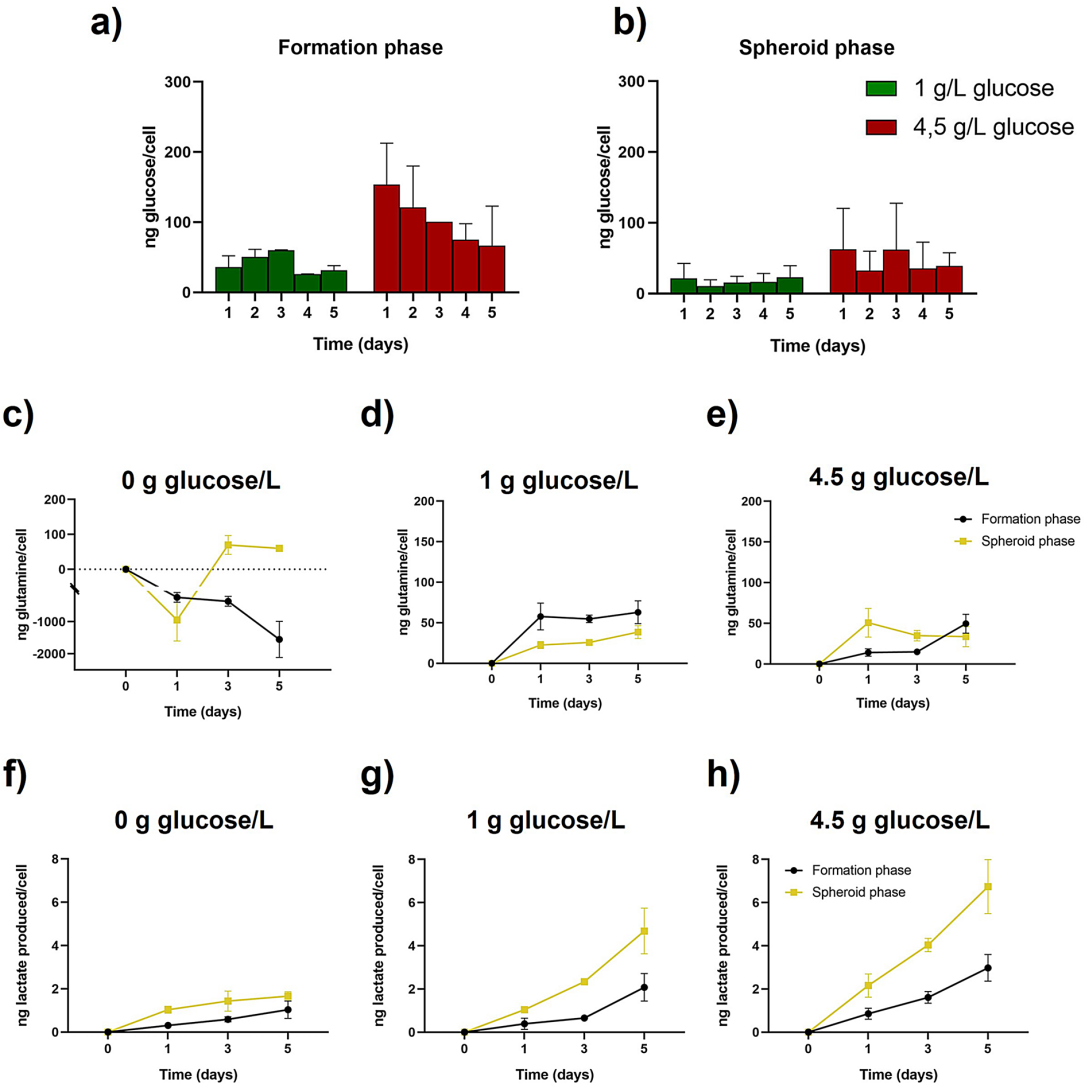
Metabolites evolution along time with cells in 3D cell culture for the A549 cell line. Glucose consumption per cell in formation (**a**) and spheroid (**b**) phase. Glutamine consumption per cell in both formation and spheroid phase for 0 g/L (**c**), 1 g/L (**d**) and 4.5 g/L (**e**) glucose. Lactate production per cell in both formation and spheroid phase for 0 g/L (**f**), 1 g/L (**g**) and 4.5 g/L (**h**) glucose.

A549 cells followed the same trend in glucose consumption as U251-MG ones in all conditions assayed, yet lower values per cell were observed (Fig. 6a, b and Table S14). Global glutamine amount in culture medium showed a clear reduction trend when glucose was present in the culture, that was reversed in the absence of glucose due to cell biosynthesis (Figure S3). Interestingly, this production shown by the negative values observed under glucose deprivation, underwent a reversal on the third day of the spheroid phase, indicating the initiation of glutamine consumption by cancer cells (Fig. 6c). In high and low glucose conditions, lung adenocarcinoma cells revealed similar behavior with respect to glutamine (Fig. 6d, e and Table S15) and lactate (Fig. 6g, h), a metabolite that cells kept producing during the entire culture time as shown by the lactate evolution in the medium (Figure S3). Even in the absence of glucose, the A549 cells still produced lactate continuously, although with a lower intensity (Fig. 6f). In this way, a direct correlation was observed between glucose availability and lactate production. See statistical analysis in Table S16.

### Inhibitors of glucose and glutamine transporters strongly affects cell proliferation and metabolites requirements

**Fig. 7:**
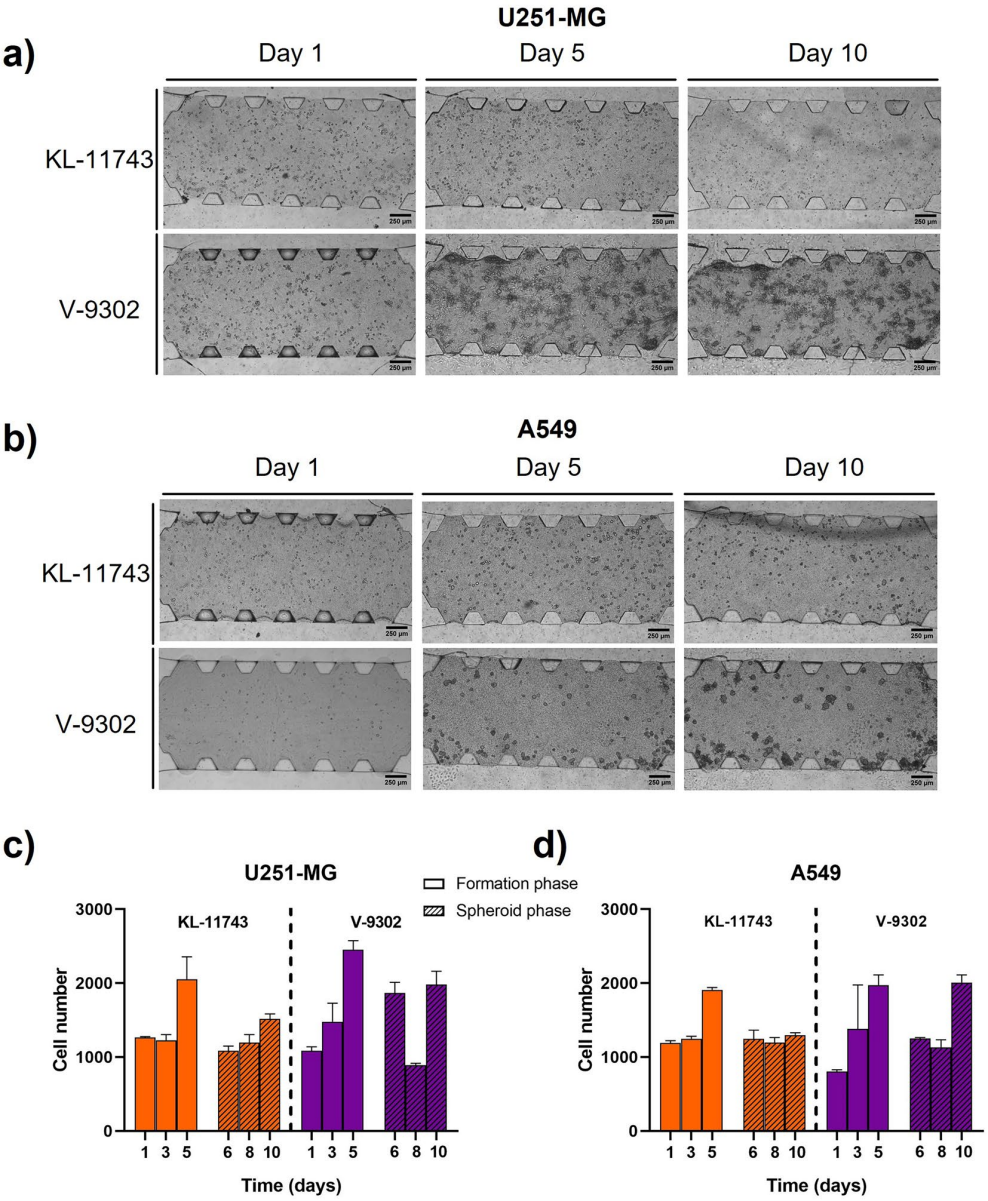
Study of the effect of KL-11743 and V-9302 inhibitors over proliferation in 3D cell culture. Brightfield images of U251-MG (**a**) and A549 (**b**) treated with both inhibitors for 10 days were taken. Quantitative data of U251-MG (**c**) and A549 (**d**) proliferation in formation and spheroid phase under the effect of KL-11743 and V-9302 inhibitors.

After characterizing cancer cell metabolism in the experiments described, we introduced KL-11743, a glucose-competitive inhibitor of the class I glucose transporters^31^, and V-9302, a competitive antagonist of transmembrane glutamine flux^32^. The aim of this assay was to evaluate whether our 3D microfluidic model was sensitive enough to detect alterations in nutrient consumption upon pharmacological inhibition of key metabolic pathways, as a way to demonstrate its potential for real-time metabolic monitoring and drug testing. A dose–response curve was performed in order to set the right concentration of drug to be used, which was 5 µM for both of them (Figure S4). A similar methodology as in the previous section was followed to study the effect of these drugs over spheroids development and metabolism. Daily monitoring of the spheroids inside the microdevice revealed a clear proliferation decrease in both cell lines, especially when KL-11743 inhibitor was used (Fig. 7a and b). Quantitative proliferation measurements obtained with Alamar Blue reagent supported these observations. Glioblastoma cells exhibited a modest increase in metabolic activity that was nearly stopped during the spheroid phase under both inhibitor treatments, with a more pronounced effect observed for the glucose transporter inhibitor (Fig. 7c). Significant differences compared to the control were particularly evident at the later stages of the experiment (Table S17). Interestingly, A549 spheroids were more affected by the treatment than U251-MG ones, revealing strong differences with respect to the control (Fig. 7d and Table S18).

**Fig. 8:**
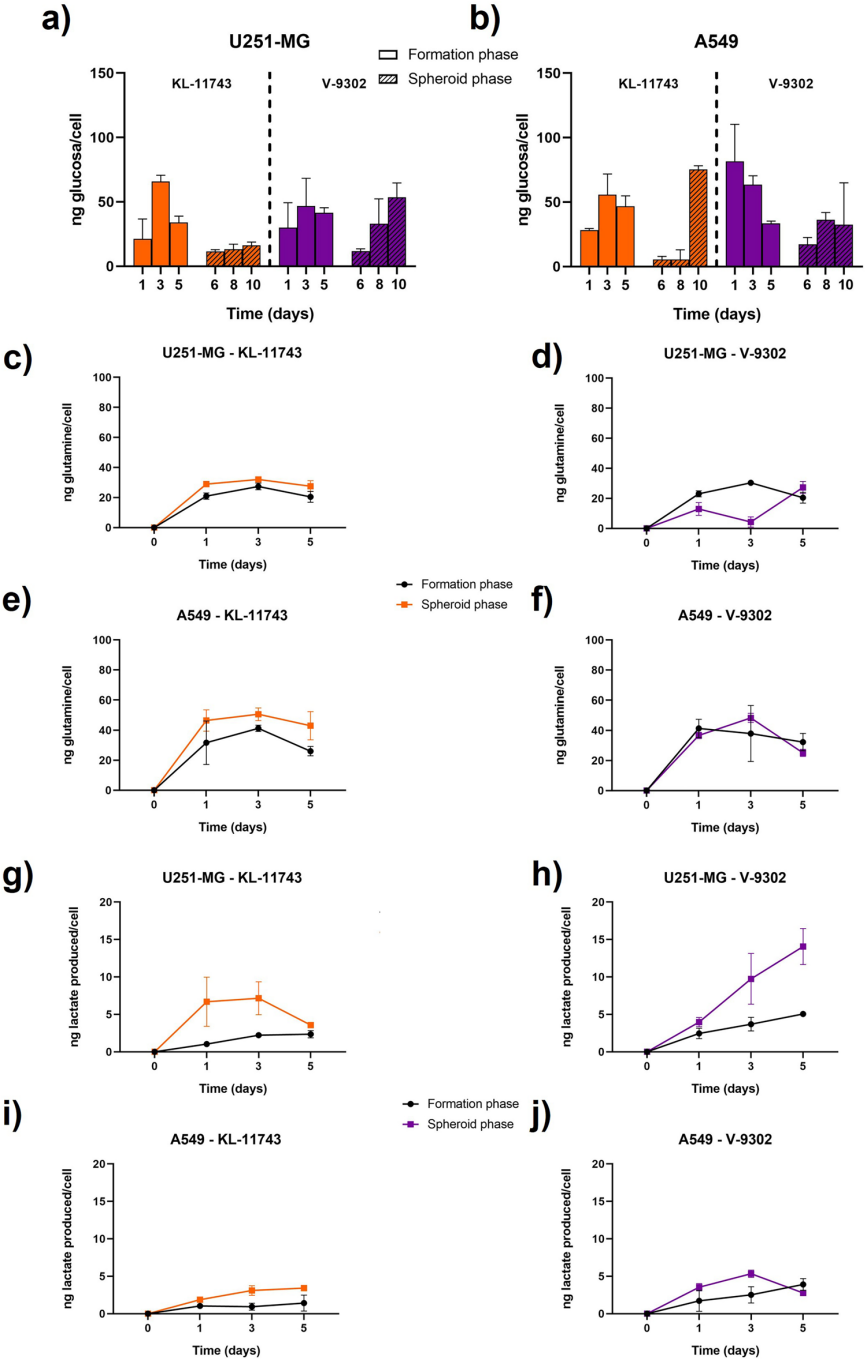
Metabolites evolution along time with cells in 3D cell culture with glucose (KL-11743) and glutamine inhibitors (V-9302). Glucose consumption per cell in formation and spheroid phases under the effect of both inhibitors for U251-MG (**a**) and A549 (**b**) cell lines. Glutamine consumption per cell in both formation and spheroid phase for U251-MG treated with KL-11743 (**c**) or with V-9302 (**d**) and for A549 (**e** and **f**, respectively). Lactate production per cell in both formation and spheroid phase for U251-MG treated with KL-11743 (**g**) or with V-9302 (**h**) and for A549 (**i** and **j**, respectively).

Although cell proliferation was clearly affected by both metabolic inhibitors, glucose consumption remained largely unchanged for both cell lines, even in the presence of the glucose transporter inhibitor (Fig. 8a and b). Interestingly, it was the glutamine transport inhibitor V-9302 that induced the most notable alteration in glucose metabolism, leading to a significant increase in glucose uptake compared to the control at day 1 in lung adenocarcinoma cells (Table S20). For U251-MG cells, whereas slight differences were observed in the glucose consumption pattern upon treatment, these changes were not statistically significant (Table S19). However, this cell line exhibited a strong decrease in glutamine consumption with both inhibitors at almost every time point (Fig. 8c and d and Table S21). In contrast, A549 spheroids displayed no significant differences in glutamine consumption with respect to the control (Fig. 8e and f and Table S22). Finally, lactate production was more affected by the glutamine transporter inhibitor than by the glucose transporter one. Glioblastoma spheroids only expressed significant differences in the spheroid phase, with V-9302 affecting the most at the latest timepoints, although KL-11743 still produced significant alterations at days 6 and 10 (Fig. 8g and h and Table S23). A549 spheroids were only affected in lactate production by the glutamine inhibitor, which produced significant disorders in both formation and spheroid phase (Fig. 8i and j and Table S24).

## Discussion

The comparison of the results obtained in 2D and 3D environments developed in this study provides interesting insights into the influence of culture model on cancer cell proliferation and metabolism. Our findings suggest that the transition from traditional 2D monolayer cultures to more physiologically relevant 3D spheroid models induce significant differences in the metabolism of cells responsible for typical tumors like glioblastoma and lung adenocarcinoma and could have implications in the development of therapeutic strategies aimed at exploiting metabolic responses. This observation aligns with previous studies comparing 2D, 3D, and in vivo xenograft models. A comprehensive metabolomic analysis of tongue cancer cell lines demonstrated that 2D cultures exhibit a more constrained metabolic profile, with significantly lower metabolite levels compared to xenografts, whereas 3D cultures more closely mimic the metabolic activity observed in tumors in vivo, including ATP production, biomass synthesis, and redox balance^33^. While 3D models still have important limitations, such as the absence of tumor microenvironment components and immune cell infiltration and vascularization, they provide a more representative metabolic landscape of tumors than conventional 2D cultures.

First, our study highlights the impact of the type of cell culture on cancer cell proliferation dynamics. Consistent with previous reports, we observed a slower proliferation rate in 3D spheroids compared to 2D monolayers^3^. This decelerated growth kinetics may be attributed to various factors, including nutrient and oxygen availability through spheroids^14^ as a consequence of a smaller interfacial area and diffusion limitations or ECM^34^, and also mechanical properties of the extracellular matrix such as porosity^35^, permeability^36^ or stiffness^37–39^. Interestingly, our results highlighted that 3D models are more independent on glucose availability than 2D ones, although proliferation and spheroid formation are also compromised in the absence of glucose. Nevertheless, our results revealed cells were able to survive and form spheroids under glucose deprivation, although the final spheroids were smaller than those formed in glucose-rich conditions. This ability to proliferate despite reduced spheroid size may be explained by changes in proteomic expression, which have been shown to be consistent across cell lines under glucose deprivation, as described by Schroll et al*.*^40^. Other studies highlight mitochondrial regulation of the unfolded protein response as a key mechanism supporting alternative metabolic pathways to ensure survival under glucose-deprived conditions^41^. Moreover, during the transition from 2 to 3D cultures, the overexpression of ALDOC and ENO2 has been identified as essential for survival in the absence of glucose^42^. Additionally, glucose deprivation has been reported to promote pseudohypoxia and dedifferentiation in lung adenocarcinoma, further underscoring the complex metabolic adaptations cells undergo in response to nutrient stress^43^. Of note, in both models, glioblastoma cells were more strongly affected when glucose was not present or removed from the culture in comparison with lung adenocarcinoma cells. This glucose dependence is consistent with the nature of glioblastoma cells as brain is an organ with high demand of this nutrient and characterized by a lipid-deprived environment^1,44^. However, lung cancer is characterized by a robust Warburg effect and multiple alternative pathways to sustain the TCA cycle independent of glucose, thereby enhancing its resistance to glucose depletion ^45,46^.

Many researchers had previously studied cancer metabolism in 3D models, mainly using techniques based on spheroid formation through cell aggregation^3,11,15,16^. Remarkably, microfluidic systems have emerged as a cutting-edge technology that offers novel insights into tumor biology, drug response, and cellular interactions within the tumor microenvironment. Many authors have reported the potential of microfluidic platforms to construct 3D tumor models that effectively mimic the architecture of the tumor microenvironment highlighting different aspects that in 2D models are missing such as tumor vascularization^47^, drug response^48^, nutrient and oxygen gradients^49^, co-culture with other stromal cells^50,51^ or ECM^52^. Our group has extensive experience in microfluidic-based models, successfully integrating key tumor microenvironment features such as extracellular matrix interactions^20,53^, and co-culture with fibroblasts^54^ and immune cells^55,56^. Additionally, we have leveraged this platform to evaluate anticancer therapies^57,58^, further demonstrating the versatility and physiological relevance of our system. One of the key advantages of our model in comparison to others is that it starts from individual cells that are embedded in a hydrogel, that acts as proxy for a physiological ECM, guiding the cell organization in a 3D spheroid. These cancer cells proliferate and assemble according to the biomechanical properties of the microenvironment, mimicking tumor formation^19^, including cell heterogeneity produced by proliferation and nutrient diffusion through spheroid^1^. Our microfluidic devices allow us to monitor tumor progression and nutrient consumption with time in a culture medium, allowing to monitor metabolic requirements in a simplified model of the tumor formation process. In contrast, most 3D studies present endpoint results, where the evolution with time of key molecules such as glucose, glutamine or lactate cannot be studied^59^. Nevertheless, our 3D microfluidic model has limitations when analyzing metabolism inside the spheroids or intracellular. Although cell recovery from our microfluidic platform is possible using collagenase digestion, allowing RNA extraction for gene expression analysis, this remains a technically challenging process. The main limitation arises from the small sample size obtained due to the scale of the device, which can impact the feasibility of downstream analyses. While we are working on optimizing this process, it remains a constraint of our system compared to other 3D models that allow for easier bulk recovery of cells for further characterization.

In this way, our results indicate that cancer cells cultured in 3D exhibit distinct metabolic profiles compared to those in 2D monolayers. This adaptation may reflect the altered microenvironment within 3D cultures, including limited nutrient diffusion and oxygen gradients, which drive cancer cells towards a less proliferative but potentially more resistant phenotype^60,61^. As described by Patra et al.^59^, global glucose consumption is much higher in 2D cell models than in 3D, but our study shows that consumption rate per cell is increased in the microfluidic device, suggesting that this difference is due to a smaller number of active cells, but with higher metabolic activity.

Glutamine consumption in a glucose-restricted environment reflects the cell’s ability to activate anaplerotic pathways, supporting essential metabolic routes such as replenishing TCA cycle intermediates, transamination reactions, GSH homeostasis, and nucleotide synthesis, all of which contribute to cell survival under adverse conditions^25^. Le et al. described the use of glutamine by cancer cells in glucose and oxygen-limited microenvironments as an essential pathway to proliferation and survival^30^, in agreement with our results in both 2D and 3D models. Similarly, Jin et al*.* demonstrated that inhibiting glutamine metabolism can induce apoptosis, as glutamine plays a crucial role in sustaining nucleotide biosynthesis, redox balance, and stemness properties in certain cancer types, ultimately influencing tumor progression and treatment resistance^32^. Interestingly, the U251-MG cell line presented a higher glutamine consumption when glucose was reduced, in agreement with literature indicating that glucose insufficiency leads to increased glutamine consumption to meet the energy requirements of brain tumors^44,62,63^. Our study aligns with these literature results, demonstrating the reliance of glioblastoma and lung adenocarcinoma on glutamine for survival under glucose deprivation. However, a key limitation of our analysis is that extracellular metabolite measurements alone do not provide a complete picture of intracellular metabolic adaptations, so the specific fate of glutamine under these conditions remains to be fully elucidated.

In addition, increased lactate production in relation with glucose concentration is an indicator of Warburg effect, corroborating the known fact that even under normoxic conditions, cancer cells degrade glucose by glycolysis^22^. This metabolite production is higher in 3D cell culture than in 2D, probably because of restrictions in nutrient and oxygen diffusion. Moreover, in the spheroid phase tumor cells tend to produce more lactate, as expected at this point of tumor development, because of hypoxic conditions produced by tumor growth^14,16^.

Finally, the inhibitors assay helped to further validate the potential of the microfluidic 3D model for real-time metabolic monitoring and therapeutic screening. The distinct responses observed between cell lines and inhibitors highlight the complexity of metabolic reprogramming and support the relevance of studying these phenomena in a dynamic 3D environment. Interestingly, despite a clear antiproliferative effect, glucose consumption remained stable in most conditions, suggesting that proliferation and nutrient uptake may not always correlate directly, as described by Han et al.^64^. This conservation of glucose uptake could potentially be explained by a compensatory activation of alternative glucose transporters not inhibited by KL-11743^31,65^. Further studies would be needed to confirm this hypothesis, including a deeper investigation into the expression patterns of glucose transporters under inhibitory conditions. The early increase in glucose consumption following glutamine inhibition may reflect a transient compensatory mechanism aimed at sustaining energy production^25,29^, similar to the glutamine increased consumption observed in our results under glucose restriction conditions. In contrast, glutamine consumption and lactate production showed cell line-specific alterations depending on the metabolic pathway inhibited, underscoring the different metabolic requirements of the cancer cell models used^44–46^. Overall, these findings reinforce the robustness of our system and its capacity to capture complex metabolic dynamics relevant for cancer research and drug development.

## Conclusions

The metabolic differences observed between 2 and 3D cultures underscore the importance of considering the spatial organization and nutrient availability in tumor microenvironments when studying cancer metabolism. In this study, we have demonstrated that cancer cell proliferation in 3D is not exponential, as seen in 2D, and we have revealed cyclic peaks of metabolic activity depending on the stage of tumor formation. In addition, our microfluidic model showed that cancer cells are able to survive for a significant time in extremely adverse microenvironments, such as no glucose supply, a condition that in 2D models results in cell death. The model and experimental design presented in this study gives valuable information regarding metabolic requirements through tumor formation and growth processes that differ from those obtained in classical 2D models. Thus, we found significant differences in glucose and glutamine consumption, as well as in lactate production. The observed changes in metabolic pathways can be used to ascertain the switch from the oxidative to the glycolytic phenotype. In conclusion, and notwithstanding their limitations, 3D models of tumor formation and cancer metabolism present a different response than classical 2D studies, and are able to provide a better analogy of the tumorigenesis process starting from individual cells to the formation and growth of a tumor spheroid.

## Materials and methods

### 2D Cell culture

Glioblastoma cell line (U251-MG) and human lung adenocarcinoma cells (A549) were obtained from the American Type Culture Collection (ATCC, USA). Cells were maintained at 37°C in a 5% CO2 humidified incubator, in Dulbecco’s modified Eagle’s medium high glucose (DMEM) (Gibco, 41966) supplemented with 10% heat-inactivated fetal bovine serum (FBS) (Gibco) and antibiotics (penicillin, streptomycin, amphotericin) (L0010, Biowest). Cells were trypsinized/passaged every 2–3 days using Trypsin 0.25% in PBS (BWSTX0915-100, Biowest). Cells were counted using a Neubauer chamber and were seeded in 35 mm diameter plates (50,000 cells per plate) using different medium according to every condition: DMEM no glucose (Gibco, 11966), DMEM low glucose–1 g/L (Gibco, 31855) or DMEM high glucose–4.5 g/L (Gibco, 41966) and incubated for 120 h. DMEM no glucose was complemented with pyruvate as same concentration than the low and high glucose media (1 mM) before treatment.

### Characterization of cell proliferation patterns in 2D culture

To characterize cell proliferation in 2D models, cells were seeded in 35 mm plates (50,000 cells per plate initially) and maintained with DMEM medium, according to every condition: high glucose–4.5 g/L, low glucose—1 g/L and no-glucose—0 g/L; until sample recovery time-point (24, 48, 72, 96 and 120 h). At every-time point cells snapshots of the plate were captured under brightfield illumination at a magnification of 4X. For quantitative measurements, at every time-point cells were washed with Phosphate buffered saline (PBS) 1 × with EDTA (Fisher Scientific, 15,443,589) twice, trypsinized and counted with a Neubauer chamber.

### Microfluidic device fabrication for 3D culture

The microfluidic systems employed consisted of Polydimethylsiloxane (PDMS) devices featuring a design encompassing a central chamber wherein a collagen-based hydrogel imitating tissue matrix is confined. Additionally, the setup involves two lateral channels for the introduction of nutrients^17^. The device structure was initially established using an SU-8 master mold on a silicon wafer, which was subsequently replicated using PDMS. This PDMS material was produced via a mixture of base and curing agent in a 10:1 weight ratio, followed by curing at 80 °C in an oven. Subsequently, the material was cropped and punched to establish access points for the channels. To affix the PDMS devices onto the glass base of 35 mm Petri dishes, surface activation through plasma treatment was performed. Subsequently, the surfaces were treated with poly-D-lysine (PDL) to enhance the adhesion of the collagen matrix to the device.

### Hydrogel preparation and cell seeding in 3D

In preparation for three-dimensional culture seeding, U251-MG and A549 cells were trypsinized, centrifuged at 1200 rpm for 5 min, and then filtered through a 40 µm cell strainer to ensure the removal of any cell aggregates. Subsequently, the cell count was determined using a Neubauer chamber, and cells were introduced into the collagen mixture to achieve a final concentration of 0.2 × 10^6^ cells/ml.

For the development of the three-dimensional cell culture within a type I collagen-based matrix, the protocol established by Shin et al.^17^ was followed. In accordance with this method, the hydrogel was created by combining 10X DPBS, collagen to reach a final concentration of 6 mg/ml, 0.5M NaOH for pH adjustment to 7.5, along with the cells. This mixture was introduced into the central chamber and allowed to polymerize at 37 °C in a humid environment. During this process, the device was rotated every 5 min for a minimum of 20 min. Finally, the hydrogel was hydrated through the lateral channels using culture medium, which will be periodically renewed. The synthesis of materials has been performed by the Platform of Production of Biomaterials and Nanoparticles of the NANBIOSIS ICTS, more specifically by the Nanoparticle Synthesis Unit of the CIBER in BioEngineering, Biomaterials & Nanomedicine (CIBER-BBN).

### 3D culture validation

**Fig. 9:**
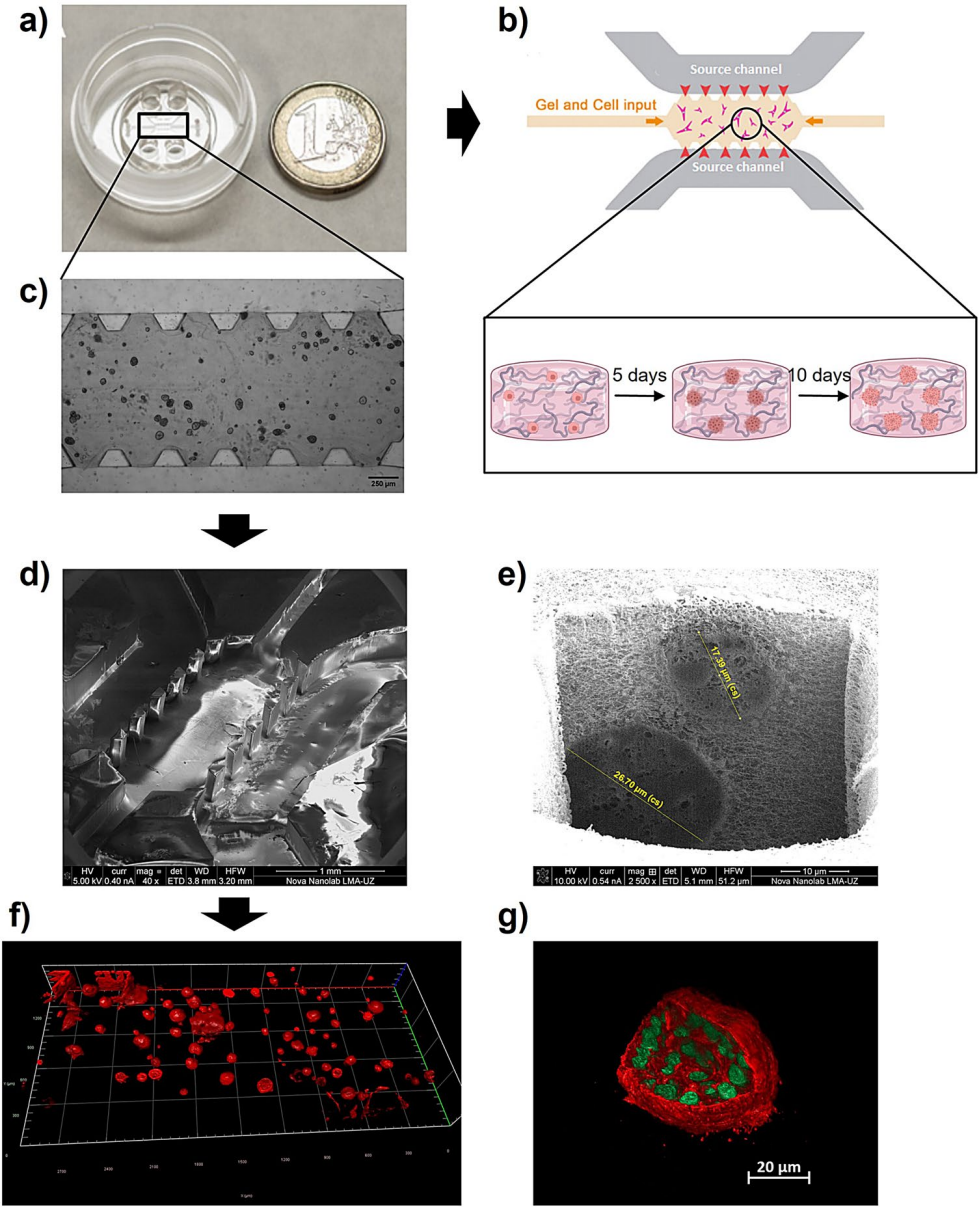
Characterization of the microfluidic 3D tumor model. (**a**) Photograph of the microfluidic device next to a 1€ coin for scale. (**b**) Schematic representation of Scheme of the central part of the device, which contained a central channel filled with collagen and embedded cell and the source and channels through which the medium is introduced. Also, a schematic representation of spheroid development over time is included. Created in BioRender. Guerrero-López, P. (2025) https://BioRender.com/98a28ll (**c**) Brightfield microscopy image showing spheroids formed in the collagen matrix inside the central chamber at day 10. SEM images of the microfluidic device, showing the structured microchannels (**d**) and a cross-section revealing spheroid organization within the collagen hydrogel (**e**). 3D reconstruction of spheroids within the central chamber of the microdevice from microscopy data (**f**) and of an individual spheroid stained with Phalloidin (cytoskeleton—red) and DAPI (nuclei—green) (**g**) using lattice light-sheet microscopy.

Once cells were seeded in the microfluidic device, various microscopy techniques were employed to validate the model and analyze cell organization within the collagen matrix. To monitor cell proliferation and spheroid formation, brightfield images were captured daily using a Leica DM IL LED microscope. Dual-beam FIB-SEM was utilized to characterize the microscopic geometry of the microfluidic device and assess spheroid organization within the collagen hydrogel. Additionally, a ZEISS Lattice Lightsheet 7 microscope at 40× magnification was used to confirm the 3D morphology and spatial arrangement of spheroids within the central chamber of the microdevice. For this purpose, spheroids were stained with Phalloidin and DAPI, followed by 3D reconstruction of both the entire central chamber and individual spheroids with higher precision. The results of the validation and schematic representations can be seen in Fig. 9.

### Characterization of cell proliferation patterns in 3D culture

For these assays, DMEM medium was used with the same glucose concentrations than in 2D assays, 4.5, 1 and 0 g/L. Culture medium was not refreshed until day 5 and then, cells were maintained for another 5 days. To characterize cell proliferation in 3D models inside the microfluidic devices under every glucose condition, spheroids growth progression was evaluated using a Leica DM IL Led microscope. Daily snapshots of the central chamber were captured under brightfield illumination at a magnification of 4X. For quantitative measurements, Alamar Blue reagent (Thermo Fisher Scientific) was used mixed with normal DMEM medium (1:9). After 4 h of incubation at 37 °C, the mixture was collected from the microdevices and its fluorescence was measured using a plate reader with a fluorescence excitation wavelength of 540–570 nm and emission at 580–610 nm. To establish the relationship between Alamar Blue fluorescence and cell number, a calibration curve was constructed across a range from 20,000 to 0 cells for each cell line, as depicted in Figure S5.

### Metabolites quantification

Culture medium was collected every day and the glucose remaining was routinely measured with a glucometer (Acofar). Each measure was repeated three times and any effect of evaporation on concentration was corrected by taking into account the variation of total volume. To validate this method, comparison with glucose concentration values obtained with Waters ACQUITY system H-Class were done, as shown in Figure S6.

Glutamine and lactate concentration in biological samples were determined in a Waters ACQUITY system H-Class UPLC, (see examples of typical chromatograms in Figure S7). For sample preparation, culture medium collected was centrifuged at 10,000 rpm for 5 min, and the supernatant was diluted 1:12.5 in a mixture of acetonitrile and Milli-Q water (50:50). The diluted samples were then filtered using 0.22 µm PTFE filters and transferred to glass vials. Specifically, glutamine was analyzed using an ACQUITY UPLC BEH Amide (130 Å, 1.7 μm; 2.1 × 50 mm, WATERS) at 85 °C. A mobile phase consisted of an initial mixture of acetonitrile/water (90:10) with 0.1% of NH_4_Cl in NH_4_OH each, at a 0.5 mL·min-1 initial flow rate increased for 2 min until a 65% acetonitrile is reached and then the system can recover initial conditions. In the case of lactate, chromatographic separation was performed using an Atlantis Premiere BEH C18AX VG (130 Å, 1.7 μm, 2.1 × 50 mm, WATERS) at 60ºC. The mobile phase employed consisted in an aqueous solution containing 0.1% of formic acid at a flow rate of 0.350 mL·min^-1^.

### Metabolites consumption calculation

Glucose and glutamine consumption and lactate production were calculated by a mass balance at the measured timepoint with respect to the initial amount in the culture medium supplied. The production or consumption rates of metabolites were normalized by dividing them by the corresponding cell number, which was determined using either Neubauer counting or Alamar Blue assay for 2D and 3D models, respectively.

The possibility of glucose sequestration by adsorption in the collagen matrix was considered, as it could interfere the calculations in 3D scenarios. To take this into account, specific adsorption assays were conducted in which culture medium with a known concentration of glucose was added to microfluidic devices containing a hydrogel with known collagen concentration but without cells. The amount of glucose adsorbed per unit mass of collagen was obtained and fitted to a Freundlich Isotherm Model. This trapped glucose was subtracted from the total glucose decrease at each time point to account only for the decrease due to cellular consumption. The same approach was applied to assess the interaction of glutamine and lactate with the collagen matrix. While glutamine showed a slight degree of adsorption (corresponding to around 1% of the total glucose present in the microchip), lactate did not exhibit measurable interaction with the gel, suggesting negligible sequestration under the tested conditions (Figure S8).

In addition, glutamine spontaneous degradation in culture medium was considered for the calculations. To this aim, we performed a kinetic curve of glutamine degradation by taking measurements over 5 days in cell-free medium (Figure S9). This allowed us to differentiate the consumption of glutamine produced by the cells from the spontaneous degradation of this metabolite.

### Inhibitors testing

The glucose transporter inhibitor KL-11743 (Sigma-Aldrich, SML3458) and the glutamine transporter one V-9302 (Sigma-Aldrich, SML3525) were tested in a dose–response curve ranging from 0 to 20 µM. For this, cells were cultured in 5 µl drops of hydrogels in a 48 well plate for 5 days in DMEM low glucose containing the drugs. After characterizing cell proliferation and glucose consumption at different concentrations, 5 µM was selected as the most suitable dose for further experiments, as it effectively modulated metabolism while preserving cell viability. The drugs were diluted in DMEM low glucose—1 g/L and added to the cells through the lateral channels of the device from the beginning of the assay, at day 0. The treatment was continuously maintained, with each medium renewal including the inhibitors.

#### Statistical analysis

Each condition underwent triplicate testing. Statistical analysis was conducted using GraphPad Prism 8 and expressed as the mean ± standard deviation (SD). Normality of the data was assessed using the Shapiro–Wilk test. When two variables were compared t-test Welch correction was performed. When more than two variables were analyzed, analysis of variance (ANOVA) was then performed, followed by post hoc Tukey tests to ascertain statistical significance across the continuous variables under different conditions. In cases where data distribution was non-normal, non-parametric Kruskal–Wallis tests were employed, followed by post hoc Dunn’s tests. All statistical tests performed are two-tailed, and a *p*-value of < 0.05 is considered significant.

## Electronic supplementary material

Below is the link to the electronic supplementary material.

Supplementary Material 1

## Data Availability

The datasets used and/or analyzed during the current study available from the corresponding author on reasonable request.

## Abbreviations

2D: Two-dimensional
3D: Three-dimensional
ECM: Extracellular matrix
DMEM: Dulbecco’s modified Eagle’s medium
FBS: Fetal bovine serum
PBS: Phosphate buffered saline
PDMS: Polydimethylsiloxane
PDL: Poly-d-lysine
TCA cycle: Tricarboxylic acid cycle
