# Supplementary material for: 2D versus 3D tumor-on-chip models to study the impact of tumor organization on metabolic patterns in vitro
Source: Sci Rep. 2025 Jun 4;15:19506. doi: 10.1038/s41598-025-03504-8 (PMC12134292; doi:10.1038/s41598-025-03504-8)
Supplement: Supplementary file 1 — Supplementary Material 1 [file 41598_2025_3504_MOESM1_ESM.docx]

Supplementary Information

**2D vs 3D Tumor-on-Chip models to study the impact of tumor organization on metabolic patterns in vitro**

Paula Guerrero-López ^a^⁑, Ana Martín-Pardillos ^b, c, d, e^⁑, Javier Bonet-Aleta ^b, c, d, f^, Andrea Mosseri^b, c, d, e^, Jose L. Hueso ^b, c, d, e, g^, Jesus Santamaria ^b, c, d, e^ and Jose Manuel Garcia-Aznar^a, e^*

*^a^* *Multiscale in Mechanical and Biological Engineering (M2BE); Aragon Institute of Engineering Research (I3A), University of Zaragoza, Mariano Esquillor s/n, 50018, Zaragoza, Spain.*

*^b^ Instituto de Nanociencia y Materiales de Aragon (INMA); CSIC-Universidad de Zaragoza, Campus Rio Ebro, Edificio I+D, C/ Poeta Mariano Esquillor, s/n, 50018, Zaragoza, Spain.*

*^c^ Department of Chemical Engineering and Environmental Technology (IQTMA), University of Zaragoza, 50018 Zaragoza, Spain.*

*^d^ Networking Research Center in Biomaterials, Bioengineering and Nanomedicine (CIBER-BBN), Instituto de Salud Carlos III; 28029 Madrid, Spain.*

*^e^ Instituto de Investigación Sanitaria (IIS) de Aragón, Avenida San Juan Bosco, 13, 50009 Zaragoza, Spain.*

*^f^* *University of Cambridge, Yusuf Hamied Department of Chemistry, Cambridge, UK CB2 1TN*

*^g^ Escuela Politécnica Superior, Universidad de Zaragoza, Crta. de Cuarte s/n, 22071, Huesca, Spain*

⁑ Both authors have contributed equally

*Corresponding author: jmgaraz@unizar.es

*
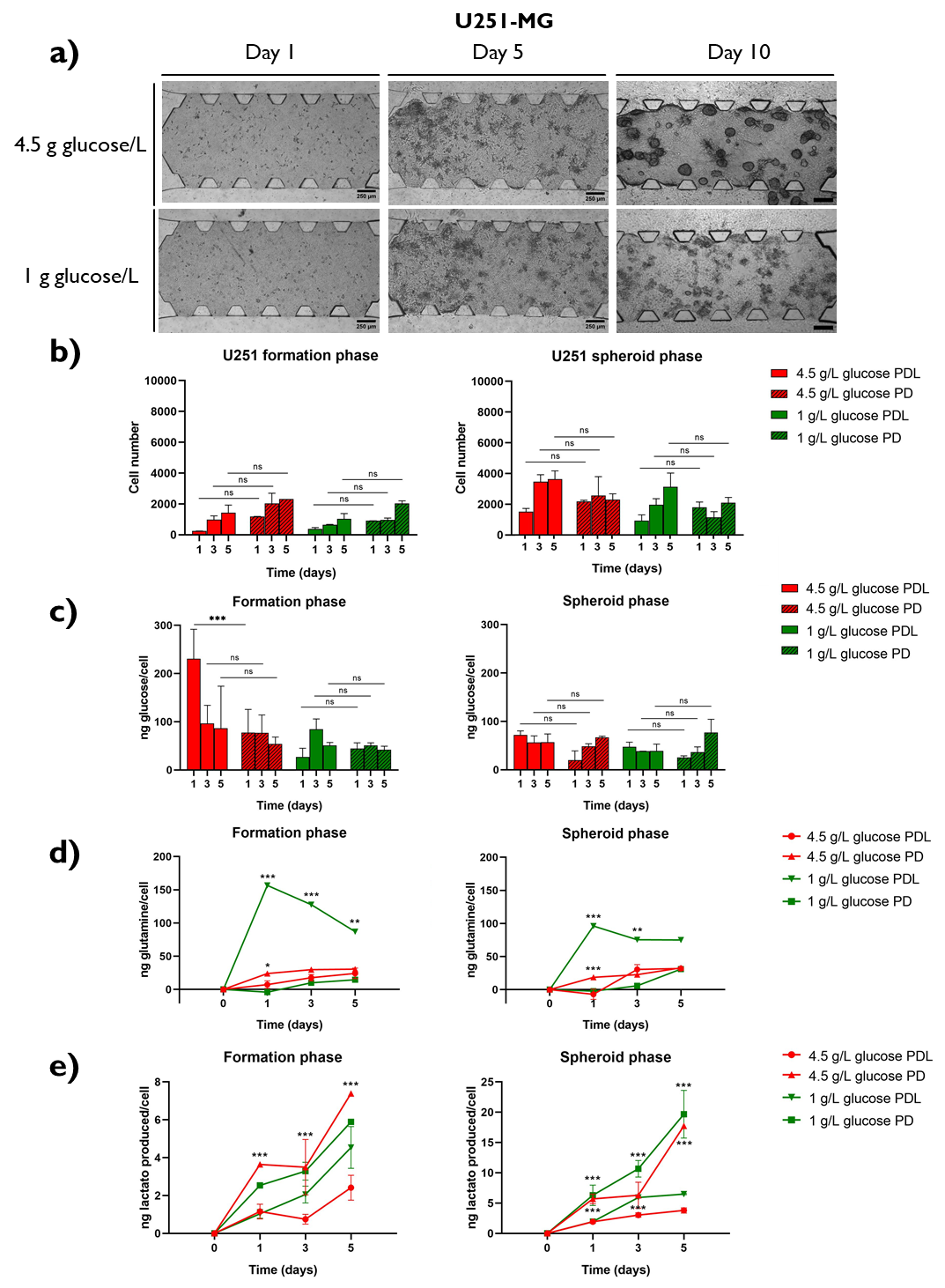
*

Supplementary Figure 1. U251-MG 3D cell culture with polydopamine (PD) coating. a) Brightfield images of U251-MG development for 10 days were taken depending on glucose availability inside the microfluidic device. Comparison of the quantitative data of U251-MG with PDL or PD coating in both formation and spheroid phase for proliferation (b), glucose consumption (c), glutamine consumption (d) and lactate production (e).


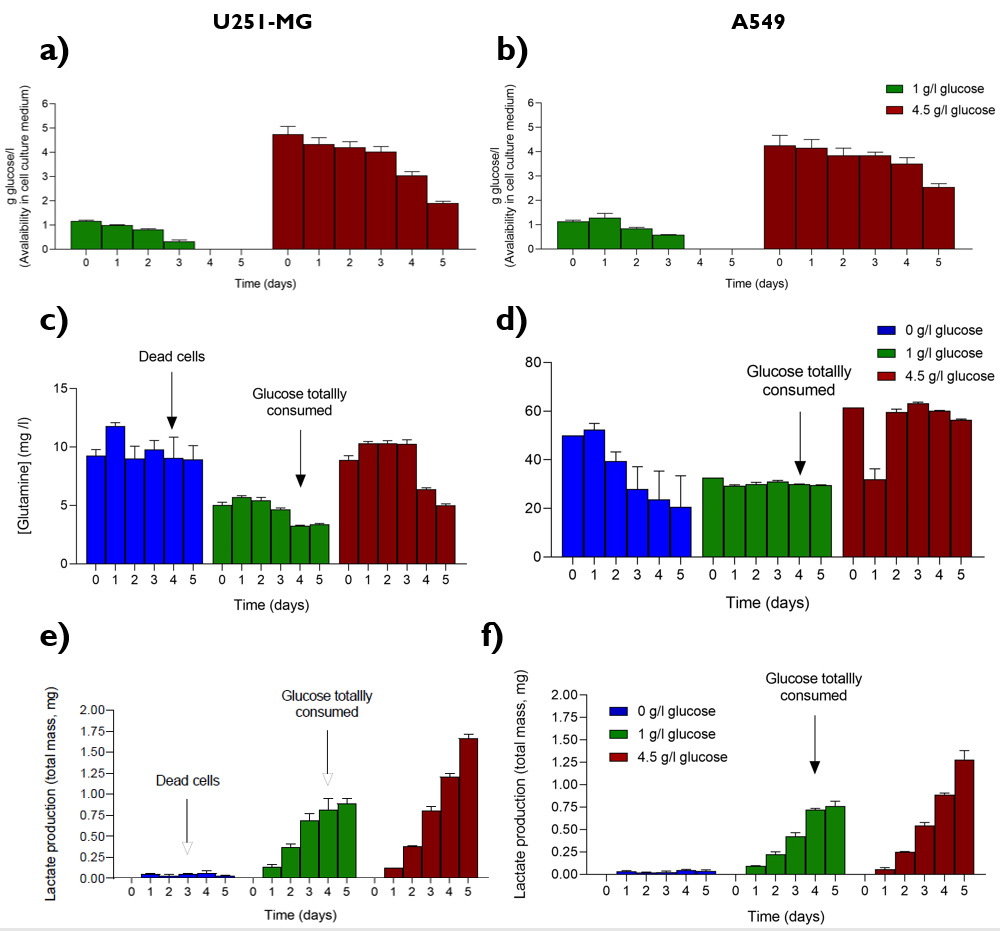


Supplementary Figure 2. Metabolites evolution in time with cells in 2D cell culture for U251-MG (left) and A549 (right) cells lines. Glucose concentration for U251-MG (a) and A549 (b). Glutamine concentration for U251-MG (c) and A549 (d). Lactate total mass for U251-MG (e) and A549 (f).


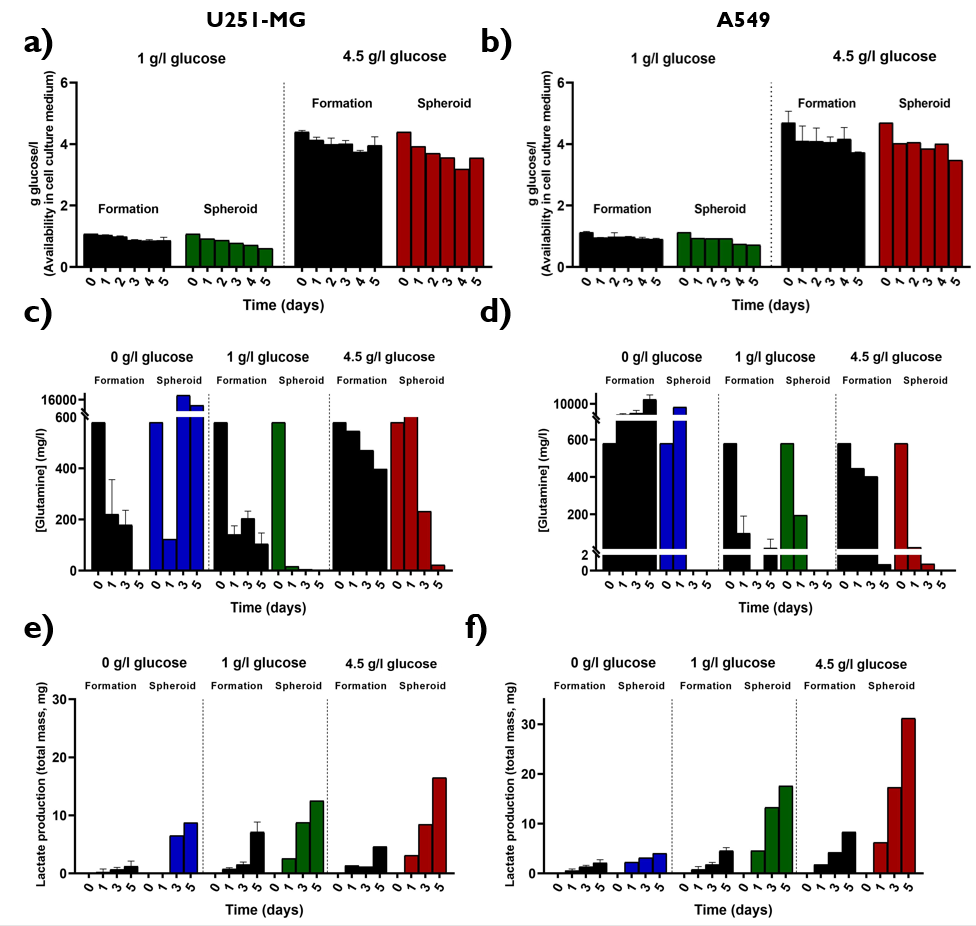


Supplementary Figure 3. Metabolites evolution in time with cells in 3D cell culture for U251-MG (left) and A549 (right) cells lines. Glucose concentration for U251-MG (a) and A549 (b). Glutamine concentration for U251-MG (c) and A549 (d). Lactate total mass for U251-MG (e) and A549 (f).


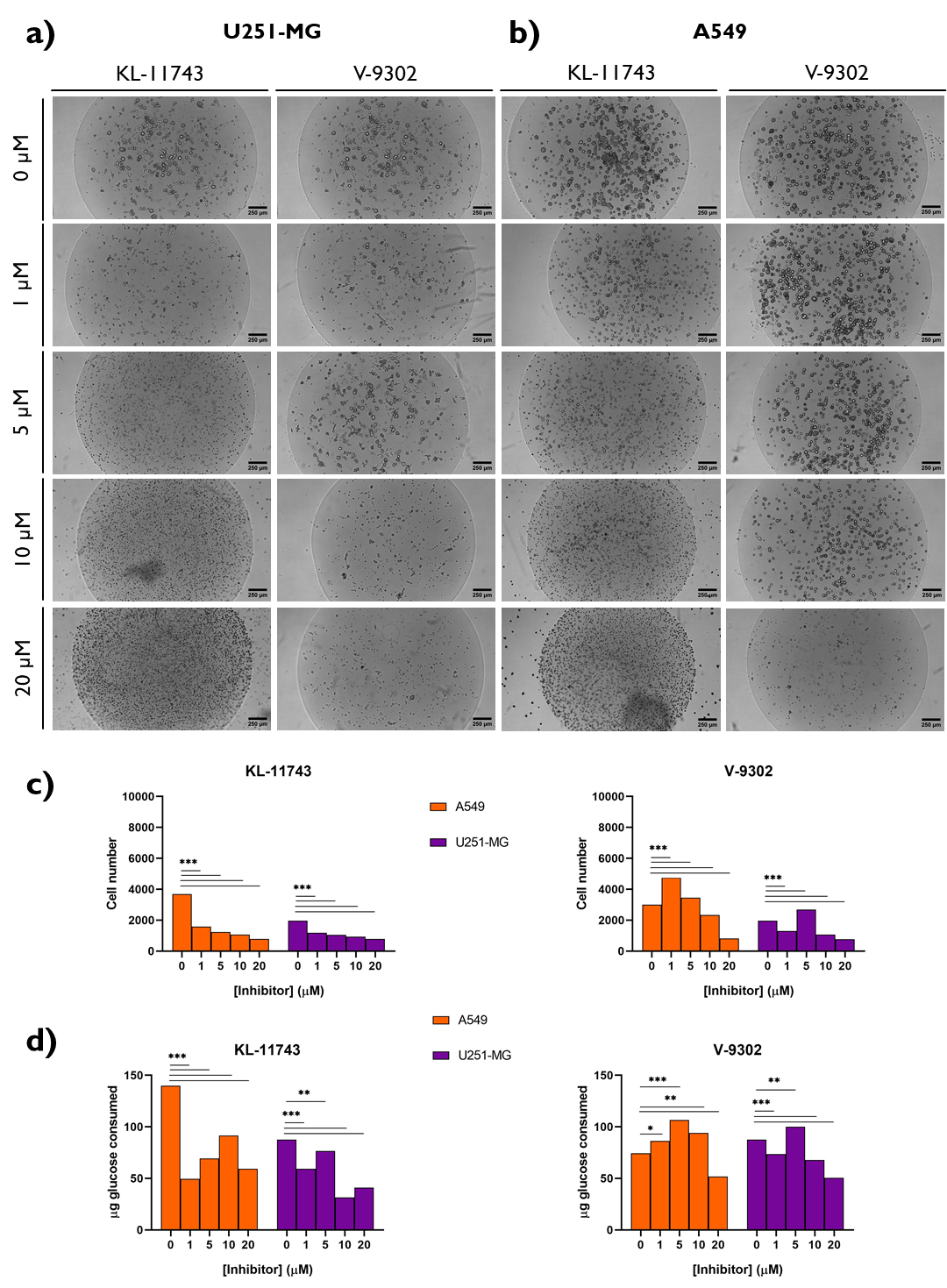
 **Supplementary Figure 4.** Dose-response curve of metabolic inhibitors KL-11743 and V-9302 on cancer cell proliferation and glucose consumption. Bright-field images of U251-MG (a) and A549 (b) at different inhibitor concentrations (0, 1, 5, 10, and 20 µM) over time. Scale bar: 250 µm. Quantification of cell proliferation in response to increasing concentrations of KL-11743 (c) and V-9302 (d). Glucose consumption in response to increasing concentrations of KL-11743 (d) and V-9302 (e). Data are presented as mean ± SD. Statistical significance is indicated as p < 0.05 (*), p < 0.01 (**), and p < 0.001 (***).


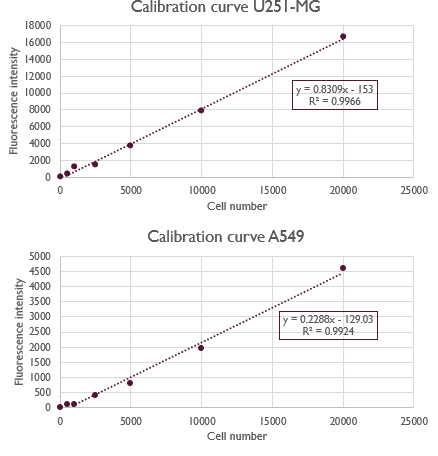


Supplementary Figure 5. Calibration curve of Alamar Blue that relates its fluorescence intensity with cell number. The calibration curve was generated using data obtained from fluorescence measurements of varying known U251-MG (up) or A549 (down) concentrations treated with Alamar Blue reagent.


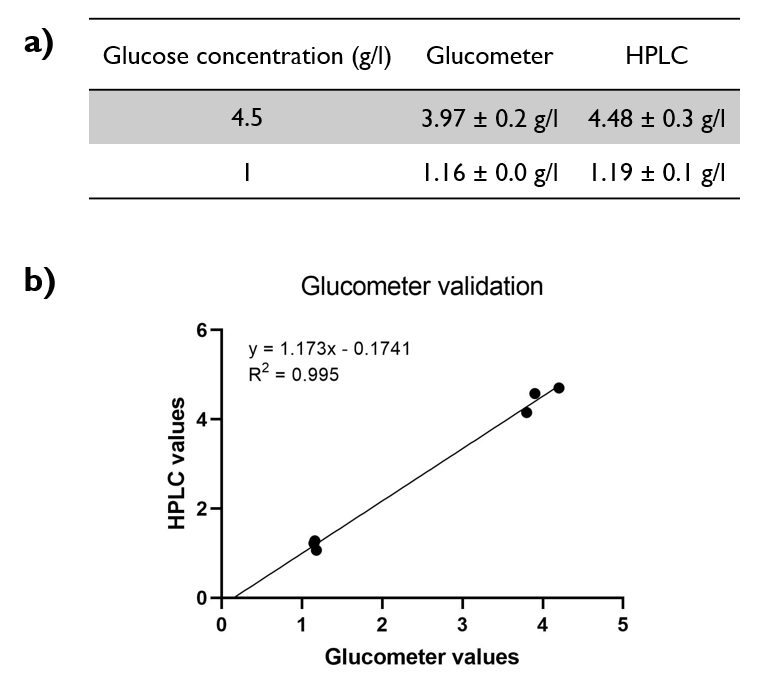


Supplementary Figure 6. Validation of the glucometer as a method for measuring glucose in the environment. The same medium sample containing 4.5 and 1 g/l glucose concentrations was analyzed using both the glucometer and the HPLC Waters ACQUITY system H-Class. a) Comparison of glucose concentration measured by the glucometer and HPLC in two different conditions: high glucose (4.5 g/L) and low glucose (1 g/L). Values are presented as mean ± standard deviation. b) Parity plot showing the correlation between glucose concentrations measured by the glucometer and HPLC. The linear regression equation and coefficient of determination (R²) indicate a strong correlation between the two measurement methods.


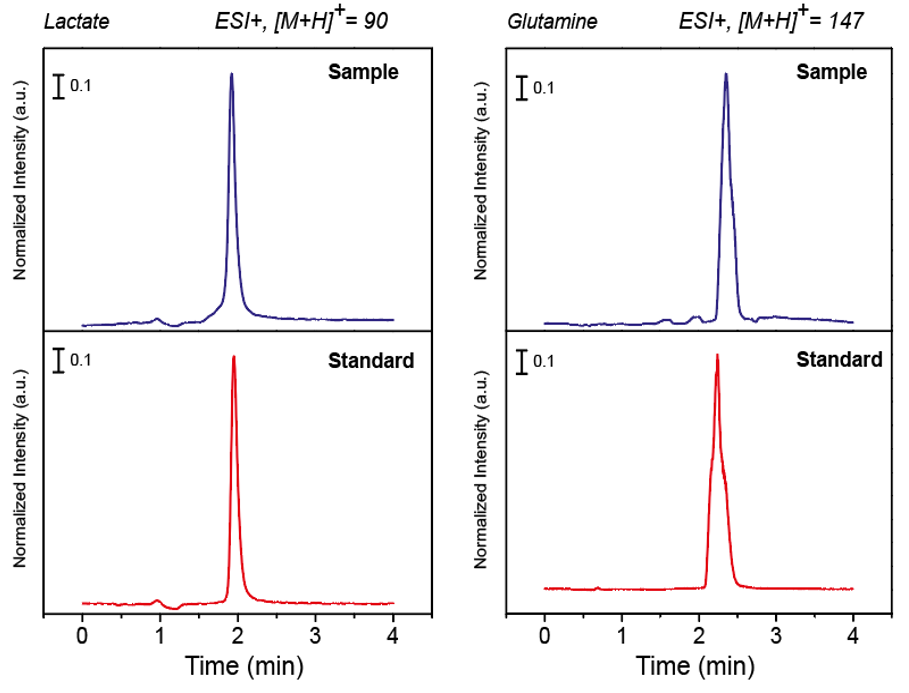


Supplementary Figure 7. Representative chromatograms of (left) lactate and (right) glutamine in samples and in standards, respectively.


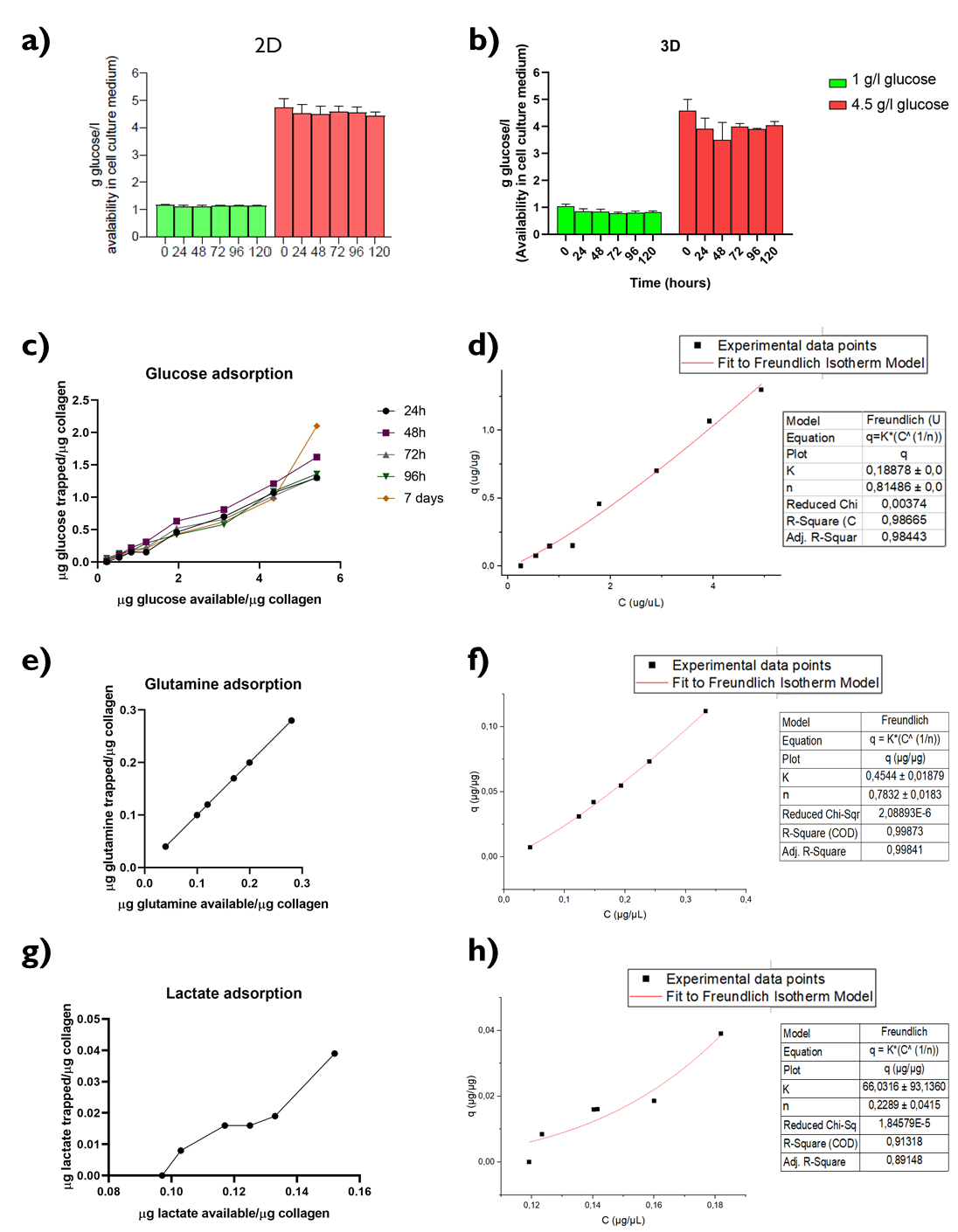


Supplementary Figure 8. Glucose concentration evolution without cells in 2D (a) and 3D (b) model system for medium with 1 and 4.5 g/l glucose. Glucose (c), glutamine (e) and lactate (g) adsorption onto collagen at different time points depending on the initial proportion of the corresponding metabolite to collagen. These data were adjusted to a Freundlich Isotherm Model for glucose (d), glutamine (f) and lactate (h).


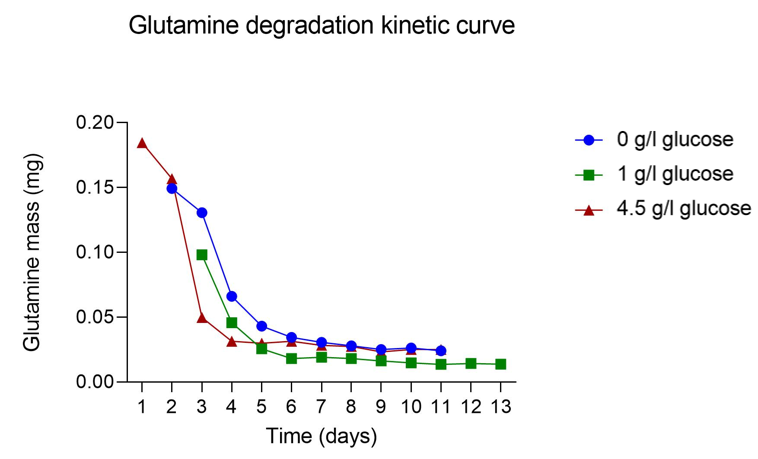
Supplementary Figure 9. Kinetic curve of glutamine spontaneous degradation in cell-free media under different glucose concentrations. The graph shows the absolute mass of glutamine (mg) every 24h in media containing 0 g/L glucose (blue circles), 1 g/L glucose (green squares), and 4.5 g/L glucose (red triangles).

| **Day** | **Condition 1** | **Condition 2** | **p-value Significance** | **p-value** |
| --- | --- | --- | --- | --- |
|  | **Glucose (g/L)** | **Glucose (g/L)** |  |  |
| **1** | 4.5 | 1 | ns | 0.5587 |
|  | 4.5 | 0 | ns | 0.5435 |
|  | 1 | 0 | ns | 0.1338 |
| **2** | 4.5 | 1 | ns | 0.8601 |
|  | 4.5 | 0 | ** | 0.0100 |
|  | 1 | 0 | * | 0.0223 |
| **3** | 4.5 | 1 | ns | 0.9968 |
|  | 4.5 | 0 | ** | 0.0020 |
|  | 1 | 0 | ** | 0.0022 |
| **4** | 4.5 | 1 | ns | 0.4706 |
|  | 4.5 | 0 | *** | 0.0003 |
|  | 1 | 0 | ** | 0.0014 |
| **5** | 4.5 | 1 | ns | 0.0519 |
|  | 4.5 | 0 | ** | 0.0016 |
|  | 1 | 0 | ns | 0.0999 |

**Supplementary Table 1.** Statistical comparisons of U251-MG proliferation under different conditions over time in 2D culture. The table presents the p-values and levels of significance for comparisons between two conditions defined by the glucose availability. Statistical significance was determined using one-way ANOVA followed by multiple comparison tests. Significance levels are indicated as follows: *p* < 0.05 (*), *p* < 0.01 (**), and *p* < 0.001 (***). Non-significant comparisons are marked as "ns".

| **Day** | **Condition 1** | **Condition 2** | **p-value Significance** | **p-value** |
| --- | --- | --- | --- | --- |
|  | **Glucose (g/L)** | **Glucose (g/L)** |  |  |
| **1** | 4.5 | 1 | * | 0.0376 |
|  | 4.5 | 0 | ** | 0.0011 |
|  | 1 | 0 | * | 0.0254 |
| **2** | 4.5 | 1 | ns | 0.8912 |
|  | 4.5 | 0 | ns | 0.1629 |
|  | 1 | 0 | ns | 0.2898 |
| **3** | 4.5 | 1 | * | 0.0271 |
|  | 4.5 | 0 | *** | 0.0005 |
|  | 1 | 0 | ** | 0.0100 |
| **4** | 4.5 | 1 | ns | 0.6947 |
|  | 4.5 | 0 | ** | 0.0047 |
|  | 1 | 0 | * | 0.0109 |
| **5** | 4.5 | 1 | * | 0.0140 |
|  | 4.5 | 0 | *** | 0.0001 |
|  | 1 | 0 | ** | 0.0019 |

**Supplementary Table 2.** Statistical comparisons of A549 proliferation under different conditions over time in 2D culture. The table presents the p-values and levels of significance for comparisons between two conditions defined by the glucose availability. Statistical significance was determined using one-way ANOVA followed by multiple comparison tests. Significance levels are indicated as follows: *p* < 0.05 (*), *p* < 0.01 (**), and *p* < 0.001 (***). Non-significant comparisons are marked as "ns".

| **Day** | **Condition 1** | | **Condition 2** | | **p-value Significance** | **p-value** |
| --- | --- | --- | --- | --- | --- | --- |
|  | **Phase** | **Glucose (g/L)** | **Phase** | **Glucose (g/L)** |  |  |
| **1** | Formation | 4.5 | Spheroid | 4.5 | ns | 0.171 |
|  | Formation | 4.5 | Formation | 1 | ns | >,999 |
|  | Formation | 4.5 | Formation | 0 | ns | >,999 |
|  | Formation | 1 | Spheroid | 1 | ns | >,999 |
|  | Formation | 1 | Formation | 0 | ns | >,999 |
|  | Formation | 0 | Spheroid | 0 | ns | >,999 |
|  | Spheroid | 4.5 | Spheroid | 1 | ns | >,999 |
|  | Spheroid | 4.5 | Spheroid | 0 | ns | 0.600 |
|  | Spheroid | 1 | Spheroid | 0 | ns | >,999 |
| **2** | Formation | 4.5 | Spheroid | 4.5 | *** | <,001 |
|  | Formation | 4.5 | Formation | 1 | ns | >,999 |
|  | Formation | 4.5 | Formation | 0 | ns | >,999 |
|  | Formation | 1 | Spheroid | 1 | *** | <,001 |
|  | Formation | 1 | Formation | 0 | ns | >,999 |
|  | Formation | 0 | Spheroid | 0 | ns | 0.977 |
|  | Spheroid | 4.5 | Spheroid | 1 | ns | >,999 |
|  | Spheroid | 4.5 | Spheroid | 0 | ns | 0.060 |
|  | Spheroid | 1 | Spheroid | 0 | ns | 0.832 |
| **3** | Formation | 4.5 | Spheroid | 4.5 | *** | <,001 |
|  | Formation | 4.5 | Formation | 1 | ns | >,999 |
|  | Formation | 4.5 | Formation | 0 | ns | >,999 |
|  | Formation | 1 | Spheroid | 1 | ns | 0.129 |
|  | Formation | 1 | Formation | 0 | ns | >,999 |
|  | Formation | 0 | Spheroid | 0 | ns | 0.968 |
|  | Spheroid | 4.5 | Spheroid | 1 | * | 0.045 |
|  | Spheroid | 4.5 | Spheroid | 0 | ** | 0.007 |
|  | Spheroid | 1 | Spheroid | 0 | ns | >,999 |
| **4** | Formation | 4.5 | Spheroid | 4.5 | * | 0.036 |
|  | Formation | 4.5 | Formation | 1 | ns | >,999 |
|  | Formation | 4.5 | Formation | 0 | ns | >,999 |
|  | Formation | 1 | Spheroid | 1 | ns | 0.921 |
| **4** | Formation | 1 | Formation | 0 | ns | >,999 |
|  | Formation | 0 | Spheroid | 0 | ns | >,999 |
|  | Spheroid | 4.5 | Spheroid | 1 | ns | 0.689 |
|  | Spheroid | 4.5 | Spheroid | 0 | ** | 0.003 |
|  | Spheroid | 1 | Spheroid | 0 | ns | 0.689 |
| **5** | Formation | 4.5 | Spheroid | 4.5 | *** | <,001 |
|  | Formation | 4.5 | Formation | 1 | ns | >,999 |
|  | Formation | 4.5 | Formation | 0 | ns | >,999 |
|  | Formation | 1 | Spheroid | 1 | *** | <,001 |
|  | Formation | 1 | Formation | 0 | ns | >,999 |
|  | Formation | 0 | Spheroid | 0 | ns | >,999 |
|  | Spheroid | 4.5 | Spheroid | 1 | ns | >,999 |
|  | Spheroid | 4.5 | Spheroid | 0 | *** | <,001 |
|  | Spheroid | 1 | Spheroid | 0 | *** | <,001 |

**Supplementary Table 3.** Statistical comparisons of U251-MG proliferation under different conditions over time in 3D culture. The table presents the p-values and levels of significance for comparisons between two conditions defined by two variables, phase and glucose condition. Statistical significance was determined using one-way ANOVA followed by multiple comparison tests. Significance levels are indicated as follows: *p* < 0.05 (*), *p* < 0.01 (**), and *p* < 0.001 (***). Non-significant comparisons are marked as "ns".

| **Day** | **Condition 1** | | **Condition 2** | | **p-value Significance** | **p-value** |
| --- | --- | --- | --- | --- | --- | --- |
|  | **Phase** | **Glucose (g/L)** | **Phase** | **Glucose (g/L)** |  |  |
| **1** | Formation | 4.5 | Spheroid | 4.5 | ns | 0.395 |
|  | Formation | 4.5 | Formation | 1 | ns | >,999 |
|  | Formation | 4.5 | Formation | 0 | ns | >,999 |
|  | Formation | 1 | Spheroid | 1 | ** | >,999 |
|  | Formation | 1 | Formation | 0 | ns | 0.005 |
|  | Formation | 0 | Spheroid | 0 | ns | 0.987 |
|  | Spheroid | 4.5 | Spheroid | 1 | ns | 0.876 |
|  | Spheroid | 4.5 | Spheroid | 0 | ns | >,999 |
|  | Spheroid | 1 | Spheroid | 0 | ns | 0.409 |
| **2** | Formation | 4.5 | Spheroid | 4.5 | * | 0.037 |
|  | Formation | 4.5 | Formation | 1 | ns | >,999 |
|  | Formation | 4.5 | Formation | 0 | ns | >,999 |
|  | Formation | 1 | Spheroid | 1 | *** | <,001 |
|  | Formation | 1 | Formation | 0 | ns | >,999 |
|  | Formation | 0 | Spheroid | 0 | ns | 0.891 |
|  | Spheroid | 4.5 | Spheroid | 1 | ** | 0.006 |
|  | Spheroid | 4.5 | Spheroid | 0 | ns | >,999 |
|  | Spheroid | 1 | Spheroid | 0 | *** | <,001 |
| **3** | Formation | 4.5 | Spheroid | 4.5 | *** | <,001 |
|  | Formation | 4.5 | Formation | 1 | ns | >,999 |
|  | Formation | 4.5 | Formation | 0 | ns | >,999 |
|  | Formation | 1 | Spheroid | 1 | *** | <,001 |
|  | Formation | 1 | Formation | 0 | ns | >,999 |
|  | Formation | 0 | Spheroid | 0 | ns | 0.982 |
|  | Spheroid | 4.5 | Spheroid | 1 | ns | >,999 |
|  | Spheroid | 4.5 | Spheroid | 0 | ** | 0.002 |
|  | Spheroid | 1 | Spheroid | 0 | * | 0.010 |
| **4** | Formation | 4.5 | Spheroid | 4.5 | *** | <,001 |
|  | Formation | 4.5 | Formation | 1 | ns | >,999 |
|  | Formation | 4.5 | Formation | 0 | ns | >,999 |
|  | Formation | 1 | Spheroid | 1 | *** | <,001 |
| **4** | Formation | 1 | Formation | 0 | ns | >,999 |
|  | Formation | 0 | Spheroid | 0 | * | 0.032 |
|  | Spheroid | 4.5 | Spheroid | 1 | ns | >,999 |
|  | Spheroid | 4.5 | Spheroid | 0 | ns | >,999 |
|  | Spheroid | 1 | Spheroid | 0 | ns | >,999 |
| **5** | Formation | 4.5 | Spheroid | 4.5 | * | >,999 |
|  | Formation | 4.5 | Formation | 1 | ns | >,999 |
|  | Formation | 4.5 | Formation | 0 | ns | 0.027 |
|  | Formation | 1 | Spheroid | 1 | *** | <,001 |
|  | Formation | 1 | Formation | 0 | ns | >,999 |
|  | Formation | 0 | Spheroid | 0 | ns | 0.963 |
|  | Spheroid | 4.5 | Spheroid | 1 | ns | >,999 |
|  | Spheroid | 4.5 | Spheroid | 0 | ns | 0.656 |
|  | Spheroid | 1 | Spheroid | 0 | * | 0.029 |

**Supplementary Table 4.** Statistical comparisons of A549 proliferation under different conditions over time in 3D culture. The table presents the p-values and levels of significance for comparisons between two conditions defined by two variables, phase and glucose condition. Statistical significance was determined using one-way ANOVA followed by multiple comparison tests. Significance levels are indicated as follows: *p* < 0.05 (*), *p* < 0.01 (**), and *p* < 0.001 (***). Non-significant comparisons are marked as "ns".

| **Day** | **Condition 1** | **Condition 2** | **p-value Significance** | **p-value** |
| --- | --- | --- | --- | --- |
|  | **Glucose (g/L)** | **Glucose (g/L)** |  |  |
| **1** | 4.5 | 1 | ns | 0.5535 |
| **2** | 4.5 | 1 | ns | 0.7205 |
| **3** | 4.5 | 1 | ns | 0.4466 |
| **4** | 4.5 | 1 | ns | 0.5808 |
| **5** | 4.5 | 1 | - | - |

**Supplementary Table 5.** Statistical comparisons of U251-MG glucose consumption under different conditions over time in 2D culture. The table presents the p-values and levels of significance for comparisons between two conditions defined by the glucose availability. Statistical significance was determined using t-test and Welch correction. Significance levels are indicated as follows: *p* < 0.05 (*), *p* < 0.01 (**), and *p* < 0.001 (***). Non-significant comparisons are marked as "ns".

| **Day** | **Condition 1** | **Condition 2** | **p-value Significance** | **p-value** |
| --- | --- | --- | --- | --- |
|  | **Glucose (g/L)** | **Glucose (g/L)** |  |  |
| **1** | 4.5 | 1 | ns | 0.1312 |
| **2** | 4.5 | 1 | ns | 0.1655 |
| **3** | 4.5 | 1 | ** | 0.0052 |
| **4** | 4.5 | 1 | ns | 0.5723 |
| **5** | 4.5 | 1 | - | - |

**Supplementary Table 6.** Statistical comparisons of A549 glucose consumption under different conditions over time in 2D culture. The table presents the p-values and levels of significance for comparisons between two conditions defined by the glucose availability. Statistical significance was determined using t-test and Welch correction. Significance levels are indicated as follows: *p* < 0.05 (*), *p* < 0.01 (**), and *p* < 0.001 (***). Non-significant comparisons are marked as "ns".

| **Day** | **Condition 1** | **Condition 2** | **p-value Significance** | **p-value** |
| --- | --- | --- | --- | --- |
|  | **Glucose (g/L)** | **Glucose (g/L)** |  |  |
| **1** | 4.5 | 1 | ns | 0.6661 |
|  | 4.5 | 0 | ns | 0.8496 |
|  | 1 | 0 | ns | 0.3806 |
| **2** | 4.5 | 1 | ns | 0.9825 |
|  | 4.5 | 0 | ns | 0.1355 |
|  | 1 | 0 | ns | 0.1702 |
| **3** | 4.5 | 1 | ns | 0.9925 |
|  | 4.5 | 0 | ns | 0.0735 |
|  | 1 | 0 | ns | 0.0852 |
| **4** | 4.5 | 1 | ns | 0.4280 |
|  | 4.5 | 0 | *** | <0,0001 |
|  | 1 | 0 | *** | <0,0001 |
| **5** | 4.5 | 1 | *** | <0,0001 |
|  | 4.5 | 0 | *** | <0,0001 |
|  | 1 | 0 | *** | <0,0001 |

**Supplementary Table 7.** Statistical comparisons of U251-MG glutamine consumption under different conditions over time in 2D culture. The table presents the p-values and levels of significance for comparisons between two conditions defined by the glucose availability. Statistical significance was determined using one-way ANOVA followed by multiple comparison tests. Significance levels are indicated as follows: *p* < 0.05 (*), *p* < 0.01 (**), and *p* < 0.001 (***). Non-significant comparisons are marked as "ns".

| **Day** | **Condition 1** | **Condition 2** | **p-value Significance** | **p-value** |
| --- | --- | --- | --- | --- |
|  | **Glucose (g/L)** | **Glucose (g/L)** |  |  |
| **1** | 4.5 | 1 | ** | 0.0084 |
|  | 4.5 | 0 | ** | 0.0023 |
|  | 1 | 0 | ns | 0.4035 |
| **2** | 4.5 | 1 | ns | 0.8528 |
|  | 4.5 | 0 | * | 0.0444 |
|  | 1 | 0 | ns | 0.0876 |
| **3** | 4.5 | 1 | ns | 0.1312 |
|  | 4.5 | 0 | *** | 0.0005 |
|  | 1 | 0 | ** | 0.0027 |
| **4** | 4.5 | 1 | ** | 0.0092 |
|  | 4.5 | 0 | *** | <0,0001 |
|  | 1 | 0 | *** | <0,0001 |
| **5** | 4.5 | 1 | ns | 0.6863 |
|  | 4.5 | 0 | *** | <0,0001 |
|  | 1 | 0 | *** | <0,0001 |

**Supplementary Table 8.** Statistical comparisons of A549 glutamine consumption under different conditions over time in 2D culture. The table presents the p-values and levels of significance for comparisons between two conditions defined by the glucose availability. Statistical significance was determined using one-way ANOVA followed by multiple comparison tests. Significance levels are indicated as follows: *p* < 0.05 (*), *p* < 0.01 (**), and *p* < 0.001 (***). Non-significant comparisons are marked as "ns".

| **Day** | **Condition 1** | **Condition 2** | **p-value Significance** | **p-value** |
| --- | --- | --- | --- | --- |
|  | **Glucose (g/L)** | **Glucose (g/L)** |  |  |
| **1** | 4.5 | 1 | ns | 0.9337 |
|  | 4.5 | 0 | ns | 0.1698 |
|  | 1 | 0 | ns | 0.2648 |
| **2** | 4.5 | 1 | ns | 0.9724 |
|  | 4.5 | 0 | ns | 0.2168 |
|  | 1 | 0 | ns | 0.1630 |
| **3** | 4.5 | 1 | ns | 0.0647 |
|  | 4.5 | 0 | *** | 0.0003 |
|  | 1 | 0 | ** | 0.0029 |
| **4** | 4.5 | 1 | ns | 0.9070 |
|  | 4.5 | 0 | ** | 0.0045 |
|  | 1 | 0 | ** | 0.0030 |
| **5** | 4.5 | 1 | ** | 0.0023 |
|  | 4.5 | 0 | *** | 0.0003 |
|  | 1 | 0 | *** | <0,0001 |

**Supplementary Table 9.** Statistical comparisons of U251-MG lactate production under different conditions over time in 2D culture. The table presents the p-values and levels of significance for comparisons between two conditions defined by the glucose availability. Statistical significance was determined using one-way ANOVA followed by multiple comparison tests. Significance levels are indicated as follows: *p* < 0.05 (*), *p* < 0.01 (**), and *p* < 0.001 (***). Non-significant comparisons are marked as "ns".

| **Day** | **Condition 1** | **Condition 2** | **p-value Significance** | **p-value** |
| --- | --- | --- | --- | --- |
|  | **Glucose (g/L)** | **Glucose (g/L)** |  |  |
| **1** | 4.5 | 1 | * | 0.0339 |
|  | 4.5 | 0 | ns | 0.9729 |
|  | 1 | 0 | * | 0.0260 |
| **2** | 4.5 | 1 | ns | 0.0694 |
|  | 4.5 | 0 | *** | 0.0003 |
|  | 1 | 0 | *** | <0,0001 |
| **3** | 4.5 | 1 | ns | 0.6256 |
|  | 4.5 | 0 | * | 0.0155 |
|  | 1 | 0 | ** | 0.0057 |
| **4** | 4.5 | 1 | * | 0.0104 |
|  | 4.5 | 0 | *** | <0,0001 |
|  | 1 | 0 | *** | <0,0001 |
| **5** | 4.5 | 1 | ns | 0.2344 |
|  | 4.5 | 0 | ** | 0.0116 |
|  | 1 | 0 | * | 0.002 |

**Supplementary Table 10.** Statistical comparisons of A549 lactate production under different conditions over time in 2D culture. The table presents the p-values and levels of significance for comparisons between two conditions defined by the glucose availability. Statistical significance was determined using one-way ANOVA followed by multiple comparison tests. Significance levels are indicated as follows: *p* < 0.05 (*), *p* < 0.01 (**), and *p* < 0.001 (***). Non-significant comparisons are marked as "ns".

| **Day** | **Condition 1** | | **Condition 2** | | **p-value Significance** | **p-value** |
| --- | --- | --- | --- | --- | --- | --- |
|  | **Phase** | **Glucose (g/L)** | **Phase** | **Glucose (g/L)** |  |  |
| **1** | Formation | 4.5 | Spheroid | 4.5 | ** | 0.003 |
|  | Formation | 4.5 | Formation | 1 | *** | <,001 |
|  | Formation | 1 | Spheroid | 1 | ns | >,999 |
|  | Spheroid | 4.5 | Spheroid | 1 | ns | >,999 |
| **2** | Formation | 4.5 | Spheroid | 4.5 | ns | 0.629 |
|  | Formation | 4.5 | Formation | 1 | ns | >,999 |
|  | Formation | 1 | Spheroid | 1 | ns | 0.714 |
|  | Spheroid | 4.5 | Spheroid | 1 | ns | >,999 |
| **3** | Formation | 4.5 | Spheroid | 4.5 | ns | 0.998 |
|  | Formation | 4.5 | Formation | 1 | ns | >,999 |
|  | Formation | 1 | Spheroid | 1 | ns | 0.988 |
|  | Spheroid | 4.5 | Spheroid | 1 | ns | >,999 |
| **4** | Formation | 4.5 | Spheroid | 4.5 | ns | >,999 |
|  | Formation | 4.5 | Formation | 1 | ns | 0.737 |
|  | Formation | 1 | Spheroid | 1 | ns | >,999 |
|  | Spheroid | 4.5 | Spheroid | 1 | ns | 0.968 |
| **5** | Formation | 4.5 | Spheroid | 4.5 | ns | >,999 |
|  | Formation | 4.5 | Formation | 1 | ns | >,999 |
|  | Formation | 1 | Spheroid | 1 | ns | >,999 |
|  | Spheroid | 4.5 | Spheroid | 1 | ns | >,999 |

**Supplementary Table 11.** Statistical comparisons of U251-MG glucose consumption under different conditions over time in 3D culture. The table presents the p-values and levels of significance for comparisons between two conditions defined by two variables, phase and glucose condition. Statistical significance was determined using one-way ANOVA followed by multiple comparison tests. Significance levels are indicated as follows: *p* < 0.05 (*), *p* < 0.01 (**), and *p* < 0.001 (***). Non-significant comparisons are marked as "ns".

| **Day** | **Condition 1** | | **Condition 2** | | **p-value Significance** | **p-value** |
| --- | --- | --- | --- | --- | --- | --- |
|  | **Phase** | **Glucose (g/L)** | **Phase** | **Glucose (g/L)** |  |  |
| **1** | Formation | 4.5 | Spheroid | 4.5 | ns | >,999 |
|  | Formation | 4.5 | Formation | 1 | *** | <,001 |
|  | Formation | 4.5 | Formation | 0 | ns | 0.220 |
|  | Formation | 1 | Spheroid | 1 | ns | >,999 |
|  | Formation | 1 | Formation | 0 | ns | >,999 |
|  | Formation | 0 | Spheroid | 0 | ns | >,999 |
|  | Spheroid | 4.5 | Spheroid | 1 | ** | 0.001 |
|  | Spheroid | 4.5 | Spheroid | 0 | ns | 0.115 |
|  | Spheroid | 1 | Spheroid | 0 | ns | >,999 |
| **3** | Formation | 4.5 | Spheroid | 4.5 | ns | >,999 |
|  | Formation | 4.5 | Formation | 1 | ** | 0.005 |
|  | Formation | 4.5 | Formation | 0 | ns | 0.745 |
|  | Formation | 1 | Spheroid | 1 | ns | >,999 |
|  | Formation | 1 | Formation | 0 | ns | >,999 |
|  | Formation | 0 | Spheroid | 0 | * | 0.018 |
|  | Spheroid | 4.5 | Spheroid | 1 | ns | >,999 |
|  | Spheroid | 4.5 | Spheroid | 0 | ns | >,999 |
|  | Spheroid | 1 | Spheroid | 0 | ** | 0.009 |
| **5** | Formation | 4.5 | Spheroid | 4.5 | ns | >,999 |
|  | Formation | 4.5 | Formation | 1 | ns | 0.374 |
|  | Formation | 4.5 | Formation | 0 | ns | 0.458 |
|  | Formation | 1 | Spheroid | 1 | ns | >,999 |
|  | Formation | 1 | Formation | 0 | ns | >,999 |
|  | Formation | 0 | Spheroid | 0 | ** | 0.002 |
|  | Spheroid | 4.5 | Spheroid | 1 | ns | >,999 |
|  | Spheroid | 4.5 | Spheroid | 0 | ns | >,999 |
|  | Spheroid | 1 | Spheroid | 0 | * | 0.047 |

**Supplementary Table 12.** Statistical comparisons of U251-MG glutamine consumption under different conditions over time in 3D culture. The table presents the p-values and levels of significance for comparisons between two conditions defined by two variables, phase and glucose condition. Statistical significance was determined using one-way ANOVA followed by multiple comparison tests. Significance levels are indicated as follows: *p* < 0.05 (*), *p* < 0.01 (**), and *p* < 0.001 (***). Non-significant comparisons are marked as "ns".

| **Day** | **Condition 1** | | **Condition 2** | | **p-value Significance** | **p-value** |
| --- | --- | --- | --- | --- | --- | --- |
|  | **Phase** | **Glucose (g/L)** | **Phase** | **Glucose (g/L)** |  |  |
| **1** | Formation | 4.5 | Spheroid | 4.5 | * | 0.046 |
|  | Formation | 4.5 | Formation | 1 | ns | >,999 |
|  | Formation | 4.5 | Formation | 0 | ns | 0.127 |
|  | Formation | 1 | Spheroid | 1 | ** | 0.004 |
|  | Formation | 1 | Formation | 0 | ns | 0.522 |
|  | Formation | 0 | Spheroid | 0 | ns | 0.947 |
|  | Spheroid | 4.5 | Spheroid | 1 | ns | >,999 |
|  | Spheroid | 4.5 | Spheroid | 0 | *** | <,001 |
|  | Spheroid | 1 | Spheroid | 0 | *** | <,001 |
| **3** | Formation | 4.5 | Spheroid | 4.5 | *** | <,001 |
|  | Formation | 4.5 | Formation | 1 | *** | <,001 |
|  | Formation | 4.5 | Formation | 0 | ns | >,999 |
|  | Formation | 1 | Spheroid | 1 | *** | <,001 |
|  | Formation | 1 | Formation | 0 | *** | <,001 |
|  | Formation | 0 | Spheroid | 0 | *** | <,001 |
|  | Spheroid | 4.5 | Spheroid | 1 | *** | <,001 |
|  | Spheroid | 4.5 | Spheroid | 0 | ns | 0.148 |
|  | Spheroid | 1 | Spheroid | 0 | *** | <,001 |
| **5** | Formation | 4.5 | Spheroid | 4.5 | *** | <,001 |
|  | Formation | 4.5 | Formation | 1 | *** | <,001 |
|  | Formation | 4.5 | Formation | 0 | *** | <,001 |
|  | Formation | 1 | Spheroid | 1 | *** | <,001 |
|  | Formation | 1 | Formation | 0 | *** | <,001 |
|  | Formation | 0 | Spheroid | 0 | *** | <,001 |
|  | Spheroid | 4.5 | Spheroid | 1 | *** | <,001 |
|  | Spheroid | 4.5 | Spheroid | 0 | ns | >,999 |
|  | Spheroid | 1 | Spheroid | 0 | *** | <,001 |

**Supplementary Table 13.** Statistical comparisons of U251-MG lactate production under different conditions over time in 3D culture. The table presents the p-values and levels of significance for comparisons between two conditions defined by two variables, phase and glucose condition. Statistical significance was determined using one-way ANOVA followed by multiple comparison tests. Significance levels are indicated as follows: *p* < 0.05 (*), *p* < 0.01 (**), and *p* < 0.001 (***). Non-significant comparisons are marked as "ns".

| **Day** | **Condition 1** | | **Condition 2** | | **p-value Significance** | **p-value** |
| --- | --- | --- | --- | --- | --- | --- |
|  | **Phase** | **Glucose (g/L)** | **Phase** | **Glucose (g/L)** |  |  |
| **1** | Formation | 4.5 | Spheroid | 4.5 | ns | 0.215 |
|  | Formation | 4.5 | Formation | 1 | * | 0.040 |
|  | Formation | 1 | Spheroid | 1 | ns | 0.988 |
|  | Spheroid | 4.5 | Spheroid | 1 | ns | 0.968 |
| **2** | Formation | 4.5 | Spheroid | 4.5 | ns | 0.239 |
|  | Formation | 4.5 | Formation | 1 | ns | 0.563 |
|  | Formation | 1 | Spheroid | 1 | ns | 0.968 |
|  | Spheroid | 4.5 | Spheroid | 1 | ns | 0.987 |
| **3** | Formation | 4.5 | Spheroid | 4.5 | ns | 0.968 |
|  | Formation | 4.5 | Formation | 1 | ns | 0.968 |
|  | Formation | 1 | Spheroid | 1 | ns | 0.955 |
|  | Spheroid | 4.5 | Spheroid | 1 | ns | 0.948 |
| **4** | Formation | 4.5 | Spheroid | 4.5 | ns | 0.968 |
|  | Formation | 4.5 | Formation | 1 | ns | 0.934 |
|  | Formation | 1 | Spheroid | 1 | ns | 0.988 |
|  | Spheroid | 4.5 | Spheroid | 1 | ns | 0.988 |
| **5** | Formation | 4.5 | Spheroid | 4.5 | ns | 0.976 |
|  | Formation | 4.5 | Formation | 1 | ns | 0.968 |
|  | Formation | 1 | Spheroid | 1 | ns | 0.988 |
|  | Spheroid | 4.5 | Spheroid | 1 | ns | 0.988 |

**Supplementary Table 14.** Statistical comparisons of A549 glucose consumption under different conditions over time in 3D culture. The table presents the p-values and levels of significance for comparisons between two conditions defined by two variables, phase and glucose condition. Statistical significance was determined using one-way ANOVA followed by multiple comparison tests. Significance levels are indicated as follows: *p* < 0.05 (*), *p* < 0.01 (**), and *p* < 0.001 (***). Non-significant comparisons are marked as "ns".

| **Day** | **Condition 1** | | **Condition 2** | | **p-value Significance** | **p-value** |
| --- | --- | --- | --- | --- | --- | --- |
|  | **Phase** | **Glucose (g/L)** | **Phase** | **Glucose (g/L)** |  |  |
| **1** | Formation | 4.5 | Spheroid | 4.5 | ns | 0.203 |
|  | Formation | 4.5 | Formation | 1 | ns | 0.067 |
|  | Formation | 4.5 | Formation | 0 | ns | >,999 |
|  | Formation | 1 | Spheroid | 1 | ns | 0.519 |
|  | Formation | 1 | Formation | 0 | ** | 0.006 |
|  | Formation | 0 | Spheroid | 0 | ns | >,999 |
|  | Spheroid | 4.5 | Spheroid | 1 | ns | >,999 |
|  | Spheroid | 4.5 | Spheroid | 0 | ** | 0.003 |
|  | Spheroid | 1 | Spheroid | 0 | ns | >,999 |
| **3** | Formation | 4.5 | Spheroid | 4.5 | ns | >,999 |
|  | Formation | 4.5 | Formation | 1 | ns | 0.067 |
|  | Formation | 4.5 | Formation | 0 | ns | >,999 |
|  | Formation | 1 | Spheroid | 1 | ns | 0.837 |
|  | Formation | 1 | Formation | 0 | ** | 0.004 |
|  | Formation | 0 | Spheroid | 0 | ** | 0.001 |
|  | Spheroid | 4.5 | Spheroid | 1 | ns | >,999 |
|  | Spheroid | 4.5 | Spheroid | 0 | ns | >,999 |
|  | Spheroid | 1 | Spheroid | 0 | ns | 0.436 |
| **5** | Formation | 4.5 | Spheroid | 4.5 | ns | >,999 |
|  | Formation | 4.5 | Formation | 1 | ns | >,999 |
|  | Formation | 4.5 | Formation | 0 | ** | 0.001 |
|  | Formation | 1 | Spheroid | 1 | ns | >,999 |
|  | Formation | 1 | Formation | 0 | *** | <,001 |
|  | Formation | 0 | Spheroid | 0 | *** | <,001 |
|  | Spheroid | 4.5 | Spheroid | 1 | ns | >,999 |
|  | Spheroid | 4.5 | Spheroid | 0 | ns | >,999 |
|  | Spheroid | 1 | Spheroid | 0 | ns | >,999 |

**Supplementary Table 15.** Statistical comparisons of A549 glutamine consumption under different conditions over time in 3D culture. The table presents the p-values and levels of significance for comparisons between two conditions defined by two variables, phase and glucose condition. Statistical significance was determined using one-way ANOVA followed by multiple comparison tests. Significance levels are indicated as follows: *p* < 0.05 (*), *p* < 0.01 (**), and *p* < 0.001 (***). Non-significant comparisons are marked as "ns".

| **Day** | **Condition 1** | | **Condition 2** | | **p-value Significance** | **p-value** |
| --- | --- | --- | --- | --- | --- | --- |
|  | **Phase** | **Glucose (g/L)** | **Phase** | **Glucose (g/L)** |  |  |
| **1** | Formation | 4.5 | Spheroid | 4.5 | *** | <,001 |
|  | Formation | 4.5 | Formation | 1 | ns | 0.403 |
|  | Formation | 4.5 | Formation | 0 | ns | 0.239 |
|  | Formation | 1 | Spheroid | 1 | ns | 0.186 |
|  | Formation | 1 | Formation | 0 | ns | 0.971 |
|  | Formation | 0 | Spheroid | 0 | ns | 0.088 |
|  | Spheroid | 4.5 | Spheroid | 1 | ** | 0.003 |
|  | Spheroid | 4.5 | Spheroid | 0 | ** | 0.003 |
|  | Spheroid | 1 | Spheroid | 0 | ns | 0.973 |
| **3** | Formation | 4.5 | Spheroid | 4.5 | *** | <,001 |
|  | Formation | 4.5 | Formation | 1 | * | 0.017 |
|  | Formation | 4.5 | Formation | 0 | ** | 0.008 |
|  | Formation | 1 | Spheroid | 1 | *** | <,001 |
|  | Formation | 1 | Formation | 0 | ns | 0.971 |
|  | Formation | 0 | Spheroid | 0 | * | 0.039 |
|  | Spheroid | 4.5 | Spheroid | 1 | *** | <,001 |
|  | Spheroid | 4.5 | Spheroid | 0 | *** | <,001 |
|  | Spheroid | 1 | Spheroid | 0 | * | 0.026 |
| **5** | Formation | 4.5 | Spheroid | 4.5 | *** | <,001 |
|  | Formation | 4.5 | Formation | 1 | * | 0.026 |
|  | Formation | 4.5 | Formation | 0 | *** | <,001 |
|  | Formation | 1 | Spheroid | 1 | *** | <,001 |
|  | Formation | 1 | Formation | 0 | ** | 0.006 |
|  | Formation | 0 | Spheroid | 0 | ns | 0.186 |
|  | Spheroid | 4.5 | Spheroid | 1 | *** | <,001 |
|  | Spheroid | 4.5 | Spheroid | 0 | *** | <,001 |
|  | Spheroid | 1 | Spheroid | 0 | *** | <,001 |

**Supplementary Table 16.** Statistical comparisons of A549 lactate production under different conditions over time in 3D culture. The table presents the p-values and levels of significance for comparisons between two conditions defined by two variables, phase and glucose condition. Statistical significance was determined using one-way ANOVA followed by multiple comparison tests. Significance levels are indicated as follows: *p* < 0.05 (*), *p* < 0.01 (**), and *p* < 0.001 (***). Non-significant comparisons are marked as "ns".

| **Day** | **Condition 1** | | **Condition 2** | | **p-value Significance** | **p-value** |
| --- | --- | --- | --- | --- | --- | --- |
|  | **Phase** | **Inhibitor** | **Phase** | **Inhibitor** |  |  |
| **1** | Formation | Control | Formation | KL - 11743 | ns | 0.075 |
|  | Formation | Control | Formation | V - 9302 | ns | 0.256 |
|  | Spheroid | Control | Spheroid | KL - 11743 | ns | >.999 |
|  | Spheroid | Control | Spheroid | V - 9302 | ns | 0.054 |
| **3** | Formation | Control | Formation | KL - 11743 | ns | 0.525 |
|  | Formation | Control | Formation | V - 9302 | ns | 0.110 |
|  | Spheroid | Control | Spheroid | KL - 11743 | ns | 0.163 |
|  | Spheroid | Control | Spheroid | V - 9302 | * | 0.017 |
| **5** | Formation | Control | Formation | KL - 11743 | * | 0.028 |
|  | Formation | Control | Formation | V - 9302 | ** | 0.001 |
|  | Spheroid | Control | Spheroid | KL - 11743 | *** | <.001 |
|  | Spheroid | Control | Spheroid | V - 9302 | * | 0.010 |

**Supplementary Table 17.** Statistical comparisons of U251-MG proliferation under different conditions over time in 3D culture. The table presents the *p*-values and levels of significance for comparisons between two conditions defined by two variables, phase and Inhibitor used. Statistical significance was determined using one-way ANOVA followed by multiple comparison tests. Significance levels are indicated as follows: *p* < 0.05 (*), *p* < 0.01 (**), and *p* < 0.001 (***). Non-significant comparisons are marked as "ns".

| **Day** | **Condition 1** | | **Condition 2** | | **p-value Significance** | **p-value** |
| --- | --- | --- | --- | --- | --- | --- |
|  | **Phase** | **Inhibitor** | **Phase** | **Inhibitor** |  |  |
| **1** | Formation | Control | Formation | KL - 11743 | ns | >.999 |
|  | Formation | Control | Formation | V - 9302 | ns | 0.845 |
|  | Spheroid | Control | Spheroid | KL - 11743 | ns | 0.700 |
|  | Spheroid | Control | Spheroid | V - 9302 | ns | 0.704 |
| **3** | Formation | Control | Formation | KL - 11743 | ns | >.999 |
|  | Formation | Control | Formation | V - 9302 | ns | >.999 |
|  | Spheroid | Control | Spheroid | KL - 11743 | *** | <.001 |
|  | Spheroid | Control | Spheroid | V - 9302 | *** | <.001 |
| **5** | Formation | Control | Formation | KL - 11743 | *** | <.001 |
|  | Formation | Control | Formation | V - 9302 | *** | <.001 |
|  | Spheroid | Control | Spheroid | KL - 11743 | *** | <.001 |
|  | Spheroid | Control | Spheroid | V - 9302 | *** | <.001 |

**Supplementary Table 18.** Statistical comparisons of A549 proliferation under different conditions over time in 3D culture. The table presents the *p*-values and levels of significance for comparisons between two conditions defined by two variables, phase and Inhibitor used. Statistical significance was determined using one-way ANOVA followed by multiple comparison tests. Significance levels are indicated as follows: *p* < 0.05 (*), *p* < 0.01 (**), and *p* < 0.001 (***). Non-significant comparisons are marked as "ns".

| **Day** | **Condition 1** | | **Condition 2** | | **p-value Significance** | **p-value** |
| --- | --- | --- | --- | --- | --- | --- |
|  | **Phase** | **Inhibitor** | **Phase** | **Inhibitor** |  |  |
| **1** | Formation | Control | Formation | KL - 11743 | ns | >.999 |
|  | Formation | Control | Formation | V - 9302 | ns | >.999 |
|  | Spheroid | Control | Spheroid | KL - 11743 | ns | 0.113 |
|  | Spheroid | Control | Spheroid | V - 9302 | ns | 0.121 |
| **3** | Formation | Control | Formation | KL - 11743 | ns | 0.866 |
|  | Formation | Control | Formation | V - 9302 | ns | 0.087 |
|  | Spheroid | Control | Spheroid | KL - 11743 | ns | 0.517 |
|  | Spheroid | Control | Spheroid | V - 9302 | ns | >.999 |
| **5** | Formation | Control | Formation | KL - 11743 | ns | 0.922 |
|  | Formation | Control | Formation | V - 9302 | ns | >.999 |
|  | Spheroid | Control | Spheroid | KL - 11743 | ns | 0.655 |
|  | Spheroid | Control | Spheroid | V - 9302 | ns | 0.975 |

**Supplementary Table 19.** Statistical comparisons of U251-MG glucose consumption under different conditions over time in 3D culture. The table presents the *p*-values and levels of significance for comparisons between two conditions defined by two variables, phase and Inhibitor used. Statistical significance was determined using one-way ANOVA followed by multiple comparison tests. Significance levels are indicated as follows: *p* < 0.05 (*), *p* < 0.01 (**), and *p* < 0.001 (***). Non-significant comparisons are marked as "ns".

| **Day** | **Condition 1** | | **Condition 2** | | **p-value Significance** | **p-value** |
| --- | --- | --- | --- | --- | --- | --- |
|  | **Phase** | **Inhibitor** | **Phase** | **Inhibitor** |  |  |
| **1** | Formation | Control | Formation | KL - 11743 | ns | >.999 |
|  | Formation | Control | Formation | V - 9302 | * | 0.036 |
|  | Spheroid | Control | Spheroid | KL - 11743 | * | 0.041 |
|  | Spheroid | Control | Spheroid | V - 9302 | ns | 0.238 |
| **3** | Formation | Control | Formation | KL - 11743 | ns | >.999 |
|  | Formation | Control | Formation | V - 9302 | ns | >.999 |
|  | Spheroid | Control | Spheroid | KL - 11743 | ns | >.999 |
|  | Spheroid | Control | Spheroid | V - 9302 | ns | 0.841 |
| **5** | Formation | Control | Formation | KL - 11743 | ns | 0.371 |
|  | Formation | Control | Formation | V - 9302 | ns | 0.953 |
|  | Spheroid | Control | Spheroid | KL - 11743 | * | 0.012 |
|  | Spheroid | Control | Spheroid | V - 9302 | ns | >.999 |

**Supplementary Table 20.** Statistical comparisons of A549 glucose consumption under different conditions over time in 3D culture. The table presents the *p*-values and levels of significance for comparisons between two conditions defined by two variables, phase and Inhibitor used. Statistical significance was determined using one-way ANOVA followed by multiple comparison tests. Significance levels are indicated as follows: *p* < 0.05 (*), *p* < 0.01 (**), and *p* < 0.001 (***). Non-significant comparisons are marked as "ns".

| **Day** | **Condition 1** | | **Condition 2** | | **p-value Significance** | **p-value** |
| --- | --- | --- | --- | --- | --- | --- |
|  | **Phase** | **Inhibitor** | **Phase** | **Inhibitor** |  |  |
| **1** | Formation | Control | Formation | KL - 11743 | *** | <.001 |
|  | Formation | Control | Formation | V - 9302 | *** | <.001 |
|  | Spheroid | Control | Spheroid | KL - 11743 | ** | 0.002 |
|  | Spheroid | Control | Spheroid | V - 9302 | *** | <.001 |
| **3** | Formation | Control | Formation | KL - 11743 | *** | <.001 |
|  | Formation | Control | Formation | V - 9302 | *** | <.001 |
|  | Spheroid | Control | Spheroid | KL - 11743 | ns | 0.123 |
|  | Spheroid | Control | Spheroid | V - 9302 | *** | <.001 |
| **5** | Formation | Control | Formation | KL - 11743 | ** | 0.002 |
|  | Formation | Control | Formation | V - 9302 | ** | 0.002 |
|  | Spheroid | Control | Spheroid | KL - 11743 | ns | 0.066 |
|  | Spheroid | Control | Spheroid | V - 9302 | ns | 0.063 |

**Supplementary Table 21.** Statistical comparisons of U251-MG glutamine consumption under different conditions over time in 3D culture. The table presents the *p*-values and levels of significance for comparisons between two conditions defined by two variables, phase and Inhibitor used. Statistical significance was determined using one-way ANOVA followed by multiple comparison tests. Significance levels are indicated as follows: *p* < 0.05 (*), *p* < 0.01 (**), and *p* < 0.001 (***). Non-significant comparisons are marked as "ns".

| **Day** | **Condition 1** | | **Condition 2** | | **p-value Significance** | **p-value** |
| --- | --- | --- | --- | --- | --- | --- |
|  | **Phase** | **Inhibitor** | **Phase** | **Inhibitor** |  |  |
| **1** | Formation | Control | Formation | KL - 11743 | * | 0.037 |
|  | Formation | Control | Formation | V - 9302 | ns | 0.497 |
|  | Spheroid | Control | Spheroid | KL - 11743 | ns | 0.071 |
|  | Spheroid | Control | Spheroid | V - 9302 | ns | 0.698 |
| **3** | Formation | Control | Formation | KL - 11743 | ns | 0.757 |
|  | Formation | Control | Formation | V - 9302 | ns | 0.455 |
|  | Spheroid | Control | Spheroid | KL - 11743 | ns | 0.054 |
|  | Spheroid | Control | Spheroid | V - 9302 | ns | 0.108 |
| **5** | Formation | Control | Formation | KL - 11743 | *** | <.001 |
|  | Formation | Control | Formation | V - 9302 | ** | 0.008 |
|  | Spheroid | Control | Spheroid | KL - 11743 | ns | >.999 |
|  | Spheroid | Control | Spheroid | V - 9302 | ns | 0.765 |

**Supplementary Table 22.** Statistical comparisons of A549 glutamine consumption under different conditions over time in 3D culture. The table presents the *p*-values and levels of significance for comparisons between two conditions defined by two variables, phase and Inhibitor used. Statistical significance was determined using one-way ANOVA followed by multiple comparison tests. Significance levels are indicated as follows: *p* < 0.05 (*), *p* < 0.01 (**), and *p* < 0.001 (***). Non-significant comparisons are marked as "ns".

| **Day** | **Condition 1** | | **Condition 2** | | **p-value Significance** | **p-value** |
| --- | --- | --- | --- | --- | --- | --- |
|  | **Phase** | **Inhibitor** | **Phase** | **Inhibitor** |  |  |
| **1** | Formation | Control | Formation | KL - 11743 | ns | >.999 |
|  | Formation | Control | Formation | V - 9302 | ns | 0.663 |
|  | Spheroid | Control | Spheroid | KL - 11743 | *** | <.001 |
|  | Spheroid | Control | Spheroid | V - 9302 | ns | 0.193 |
| **3** | Formation | Control | Formation | KL - 11743 | ns | >.999 |
|  | Formation | Control | Formation | V - 9302 | ns | 0.461 |
|  | Spheroid | Control | Spheroid | KL - 11743 | ns | 0.859 |
|  | Spheroid | Control | Spheroid | V - 9302 | *** | <.001 |
| **5** | Formation | Control | Formation | KL - 11743 | ns | 0.130 |
|  | Formation | Control | Formation | V - 9302 | ns | >.999 |
|  | Spheroid | Control | Spheroid | KL - 11743 | * | 0.012 |
|  | Spheroid | Control | Spheroid | V - 9302 | *** | <.001 |

**Supplementary Table 23.** Statistical comparisons of U251-MG lactate production under different conditions over time in 3D culture. The table presents the *p*-values and levels of significance for comparisons between two conditions defined by two variables, phase and Inhibitor used. Statistical significance was determined using one-way ANOVA followed by multiple comparison tests. Significance levels are indicated as follows: *p* < 0.05 (*), *p* < 0.01 (**), and *p* < 0.001 (***). Non-significant comparisons are marked as "ns".

| **Day** | **Condition 1** | | **Condition 2** | | **p-value Significance** | **p-value** |
| --- | --- | --- | --- | --- | --- | --- |
|  | **Phase** | **Inhibitor** | **Phase** | **Inhibitor** |  |  |
| **1** | Formation | Control | Formation | KL - 11743 | ns | >.999 |
|  | Formation | Control | Formation | V - 9302 | ns | 0.663 |
|  | Spheroid | Control | Spheroid | KL - 11743 | ns | <.001 |
|  | Spheroid | Control | Spheroid | V - 9302 | * | 0.193 |
| **3** | Formation | Control | Formation | KL - 11743 | ns | >.999 |
|  | Formation | Control | Formation | V - 9302 | *** | 0.461 |
|  | Spheroid | Control | Spheroid | KL - 11743 | ns | 0.859 |
|  | Spheroid | Control | Spheroid | V - 9302 | ns | <.001 |
| **5** | Formation | Control | Formation | KL - 11743 | ns | 0.130 |
|  | Formation | Control | Formation | V - 9302 | *** | >.999 |
|  | Spheroid | Control | Spheroid | KL - 11743 | ns | 0.012 |
|  | Spheroid | Control | Spheroid | V - 9302 | ** | <.001 |

**Supplementary Table 24.** Statistical comparisons of A549 lactate production under different conditions over time in 3D culture. The table presents the *p*-values and levels of significance for comparisons between two conditions defined by two variables, phase and Inhibitor used. Statistical significance was determined using one-way ANOVA followed by multiple comparison tests. Significance levels are indicated as follows: *p* < 0.05 (*), *p* < 0.01 (**), and *p* < 0.001 (***). Non-significant comparisons are marked as "ns".
